# Heterologous expression of influenza haemagglutinin leads to early and transient activation of the unfolded protein response in *Nicotiana benthamiana*

**DOI:** 10.1111/pbi.14252

**Published:** 2023-12-01

**Authors:** Louis‐Philippe Hamel, Marc‐André Comeau, Rachel Tardif, Francis Poirier‐Gravel, Marie‐Ève Paré, Pierre‐Olivier Lavoie, Marie‐Claire Goulet, Dominique Michaud, Marc‐André D'Aoust

**Affiliations:** ^1^ Medicago Inc. Québec Quebec Canada; ^2^ Centre de recherche et d'innovation sur les végétaux, Département de phytologie Université Laval Québec Quebec Canada

**Keywords:** unfolded protein response, endoplasmic reticulum stress, transient *Agrobacterium*‐mediated expression, influenza haemagglutinin, virus‐like particles, plant molecular farming

## Abstract

The unfolded protein response (UPR) allows cells to cope with endoplasmic reticulum (ER) stress induced by accumulation of misfolded proteins in the ER. Due to its sensitivity to *Agrobacterium tumefaciens*, the model plant *Nicotiana benthamiana* is widely employed for transient expression of recombinant proteins of biopharmaceutical interest, including antibodies and virus surface proteins used for vaccine production. As such, study of the plant UPR is of practical significance, since enforced expression of complex secreted proteins often results in ER stress. After 6 days of expression, we recently reported that influenza haemagglutinin H5 induces accumulation of UPR proteins. Since up‐regulation of corresponding UPR genes was not detected at this time, accumulation of UPR proteins was hypothesized to be independent of transcriptional induction, or associated with early but transient UPR gene up‐regulation. Using time course sampling, we here show that H5 expression does result in early and transient activation of the UPR, as inferred from unconventional splicing of *NbbZIP60* transcripts and induction of UPR genes with varied functions. Transient nature of H5‐induced UPR suggests that this response was sufficient to cope with ER stress provoked by expression of the secreted protein, as opposed to an antibody that triggered stronger and more sustained UPR activation. As up‐regulation of defence genes responding to H5 expression was detected after the peak of UPR activation and correlated with high increase in H5 protein accumulation, we hypothesize that these immune responses, rather than the UPR, were responsible for onset of the necrotic symptoms on H5‐expressing leaves.

## Graphical abstract

*Agrobacterium*‐mediated expression of influenza haemagglutinins allows for the production of plant‐made vaccines via accumulation of virus‐like particles. In plant cells biofactories, this molecular farming process results in early and transient activation of the unfolded protein response, which likely favors recombinant protein expression.

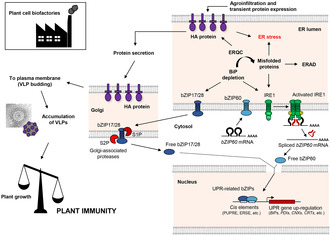

## Introduction

In eukaryotes, many proteins rely on the cell secretory pathway for proper maturation, targeting, and biological functions (Benham, 2012). These include secreted proteins, plasma membrane (PM) proteins, and proteins targeted to lysosomes (and the vacuole in plant cells). To enter the secretory pathway, proteins are first translated on membrane‐bound ribosomes located on the external face of the ER. Nascent polypeptides then reach luminal space of the ER via translocons that act as protein channels spanning the ER membrane. Inside the ER lumen, the so‐called ‘client’ proteins are rapidly subjected to chaperone‐assisted folding and, when required, to post‐translational modifications such as disulfide bridge formation, glycosylation, or oligomer assembly. To coordinate these activities, eukaryotic cells evolved a sophisticated protein machinery known as the endoplasmic reticulum quality control (ERQC) system (Araki and Nagata, 2011). While protein disulfide isomerases (PDIs) catalyse the formation of disulfide bonds, ER‐resident chaperones of the binding immunoglobulin protein (BiP) family bind to exposed hydrophobic regions of unfolded proteins, helping them to adopt a proper tertiary structure. For glycoproteins, folding assistance is further provided by ER‐resident lectins of the calreticulin (CRT) and calnexin (CNX) families.

Endoplasmic reticulum quality control allows for proper folding and modification of most client proteins, which in turn proceed towards downstream steps of the secretory pathway for further maturation, suitable targeting, or secretion. Despite efficacy of the ERQC system, some client proteins still fail to fold or assemble properly. When accumulation of misfolded proteins reaches a certain threshold, a second mechanism known as the ER‐associated degradation (ERAD) system is activated (Araki and Nagata, 2011). ERAD allows for the retro‐transport of misfolded proteins from the ER lumen to the cytosol, where degradation through the 26S proteasome will intervene. Distinct ERAD pathways exist (Brodsky and Wojcikiewicz, 2009; Wu and Rapoport, 2018); however, the removal of soluble and ER membrane glycoproteins is mediated by luminal lectin osteosarcoma 9 (OS9; Hüttner *et al*., 2012), which recognizes trimmed N‐glycan chains of glycoproteins that have failed to fold properly (Liu and Howell, 2010). Together with the ER‐resident chaperone glucose‐regulated protein 94 (GRP94), OS9 brings misfolded proteins to the retrotranslocation complex, which comprises ER membrane proteins such as Sel1L, HRD1, and Derlins (DERs). The retrotranslocation complex also comprises ATPase motor protein CDC48, which extracts misfolded proteins from the ER lumen and releases them in the cytosol. E3 ubiquitin ligase activity of HRD1 then conjugates ubiquitin moieties to the target protein, marking it for degradation.

While ERQC and ERAD constantly function to maintain cell homeostasis, changes in environmental conditions or cell physiological status can increase the needs for protein secretion. These changes can also induce conditions that are no longer favourable for folding and maturation of ER proteins. When accumulation of misfolded proteins overwhelms basal ERQC and ERAD functions, cells start to experience ER stress. To cope with ER stress, eukaryotes have evolved refined signalling networks collectively known as the unfolded protein response (UPR; Read and Schröder, 2021). In plants, the UPR comprises two branches with conserved components and activation mechanisms (Duwi Fanata *et al*., 2013; Howell, 2013, 2021; Iwata and Koizumi, 2012; Liu and Howell, 2010). In the first branch, membrane‐tethered transcription factors (TFs) basic leucine zipper 17 (bZIP17) and bZIP28 are released from membrane anchoring via clipping of their transmembrane domain (TMD). This process is mediated by proteases associated with the Golgi apparatus (Liu *et al*., 2007). In the second branch, the ER transmembrane sensor inositol requiring enzyme 1 (IRE1) uses its ribonuclease activity to unconventionally splice *bZIP60* transcripts, producing a shorter TF that lacks a TMD and therefore is no longer restrained by ER membrane anchoring (Nagashima *et al*., 2011). Once activated, the UPR triggers a series of complementary mechanisms, including the shutdown of translational activities and the up‐regulation of UPR genes. These include ERQC components such *PDIs*, *BiPs*, *CNXs*, and *CRTs*, as well as genes encoding ERAD components (Iwata *et al*., 2008, 2010; Kamauchi *et al*., 2005). The purpose of the UPR is to restore cell homeostasis by reducing translation on one hand, and increasing ERQC and ERAD capabilities on the other hand. In case of severe ER stress, sustained activation of the UPR can also lead to the activation of programmed cell death, a mechanism that ultimately protects highly stressed tissues from cells that have become dysfunctional (Kørner *et al*., 2015).

Plant molecular farming collectively refers to approaches that make use of plant cells as biofactories to produce recombinant proteins or metabolites of biopharmaceutical interest (Chung *et al*., 2022). Recombinant proteins commonly produced *in planta* include therapeutic antibodies as well as surface proteins from mammalian viruses that are used for the production of vaccines. Using *Nicotiana benthamiana* leaf cells, the biopharmaceutical company Medicago has for instance developed a molecular farming approach to produce influenza vaccine candidates at large scale (D'Aoust *et al*., 2008; Landry *et al*., 2010). Based on the bacterial vector *Rhizobium radiobacter* (commonly known and hereafter referred to as *Agrobacterium tumefaciens*), this process relies on the transient expression of recombinant influenza haemagglutinins (HAs), along with the viral suppressor of RNA silencing P19 (Silhavy *et al*., 2002) that prevents silencing of recombinant *HA* genes delivered by the bacterium. Engineered to efficiently enter the ER, newly synthesized HA proteins travel through the plant cell secretory pathway, before being trafficked to the PM (D'Aoust *et al*., 2008). When sufficient HA proteins have accumulated, bending of the PM allows for budding of the so‐called virus‐like particles (VLPs). These nanoscale assemblies comprise trimer clusters of the engineered HA protein embedded in a lipid envelope derived from the PM of plant cells. Structurally, VLPs and influenza viruses share similar size and shape; however, the former lack other viral proteins such as the surface exposed neuraminidase, in addition to being devoid of the genetic components required for replication. Once purified and formulated into vaccine candidates, VLPs induce an immune response that protects newly immunized hosts from subsequent infection by the influenza virus (Landry *et al*., 2010).

Using influenza HA protein H5, we recently reported that expression of VLPs results in a unique molecular signature that affects metabolism and fitness of plant cells at 6 days post‐infiltration (DPI; Hamel *et al*., 2023). In addition to the shutdown of chloroplast gene expression and to the activation of immune pathways, proteomics revealed that H5 expression results in the accumulation of UPR proteins, including PDIs, BiPs, and CRTs. Enforced expression of a complex secreted protein such as H5 was expected to trigger the UPR so that plant cells can manage stress associated to the transient expression system, including increased needs for both recombinant and endogenous defence protein secretion. Interestingly, thorough monitoring of the transcriptome at 6 DPI did not reveal induction of UPR genes, suggesting that increased accumulation of UPR proteins was either independent of transcriptional regulation, or that UPR gene up‐regulation occurred earlier, before returning to levels that prevented their detection at 6 DPI (Hamel *et al*., 2023).

Using time course sampling, we here show that H5 expression does result in early and transient activation of the UPR, as inferred from the detection of unconventional splicing of *NbbZIP60* transcripts and the up‐regulation of UPR genes with varied functions. Up‐regulation of UPR genes was detectable at 3 DPI, but expression of these genes had returned to basal level after 5 days of expression. Our data also showed that activation of the UPR peaked prior to the induction of defence genes that strongly respond to H5 protein expression. Transient nature of the H5‐induced UPR suggests that enhanced ERQC and ERAD functions were sufficient to cope with ER stress imposed by expression of the HA protein, as opposed to the expression of an antibody that induced stronger and more sustained activation of the UPR. Overall, this work expands our understanding of host–plant responses to foreign protein expression, in addition of providing the research community with a useful set of marker genes to study ER stress and associated UPR in *N. benthamiana*, a model plant and host of choice for molecular farming and study of plant immunity (Bally *et al*., 2018; Chung *et al*., 2022; Goodin *et al*., 2008; Ranawaka *et al*., 2023). Conserved molecular mechanisms linked to the activation of UPR in *N. benthamiana* are further discussed.

## Results

### Accumulation of UPR proteins in response to H5 expression

To better define molecular responses in *N. benthamiana* leaves expressing influenza VLPs, we previously conducted a proteomics survey using isobaric tags for relative and absolute quantitation (iTRAQ) labelling (Hamel *et al*., 2023). At 6 DPI, this revealed enhanced accumulation of UPR proteins, including PDIs, BiPs, and CRTs (Table 1). Since the UPR plays a central role in protein secretion, we further investigated regulation of this pathway during foreign protein expression. Search of the *N. benthamiana* genome identified 21 PDIs (NbPDIs) that clustered similarly to PDIs from the model plant species *Arabidopsis thaliana* (AtPDIs; Figure S1a). A list providing identification numbers from all UPR genes identified is available in the Supporting information section (Table S1). Of the seven NbPDIs identified by proteomics, all but NbPDI16 and NbPDI17 displayed a C‐terminal ‘KDEL’ motif (Table 1), which works as an ER retention signal (Munro and Pelham, 1987). Interestingly, identified NbPDIs also matched with the subset of AtPDIs previously involved in the UPR (Lu and Christopher, 2008), including NbPDI16 and NbPDI17 that lack an ER retention signal (Figure S1a). Search of the *N. benthamiana* genome also identified seven BiPs (NbBiPs; Figure S1b), and nine ER lectins that clustered in two sub‐types corresponding to CRTs (NbCRTs) and CNXs (NbCNXs; Figure S1c). For these ER‐resident chaperones, clustering was again similar to corresponding homologues in Arabidopsis, emphasizing conservation of ERQC components between the two plant species. For all NbBiPs and NbCRTs identified by proteomics, a C‐terminal ‘HDEL’ motif was identified, again suggesting retention in the ER (Table 1).

**Table 1:** UPR proteins up‐regulated by P19 expression or co‐expression of P19 and influenza H5.

UniProt No	Annotation	P19 vs Mock		H5 vs Mock		Best corresponding gene model (Niben No)	ER retention signal	Sequences of the last 25 amino acids (total predicted number of amino acids)	Attributed gene name
		Log2FC	*Z* score	Log2FC	*Z* score				
K4C2W4	Protein disulfide‐isomerase OS=*Solanum lycopersicum*	0.97	0.95	1.90	1.66	Niben101Scf01594g05014	Yes	…DKSAQSEADSTTSESVNTDSAKDEL• (503)	*NbPDI8*
M0ZYC1	Protein disulfide‐isomerase OS=*Solanum tuberosum*	1.28	1.32	1.81	1.57	Niben101Scf04582g03004	Yes	…EFIEKNRDKPVQSDSARTDSAKDEL• (509)	*NbPDI13*
K4BRS2	Protein disulfide‐isomerase OS=*Solanum lycopersicum*	1.01	1.00	1.68	1.44	Niben101Scf05405g08004	Yes	…STQKPTEFDANSSPESTIDDVKDEL• (571)	*NbPDI1*
M1AZ99	Protein disulfide‐isomerase OS=*Solanum tuberosum*	1.07	1.08	1.65	1.42	Niben101Scf02827g08001	Yes	…STQKPTEFDANSSPESTIDDVKDEL• (571)	*NbPDI2*
P93358	Protein disulfide‐isomerase OS=*Nicotiana tabacum*	0.85	0.81	1.45	1.22	Niben101Scf00466g04033	No	…MLAKSISQAKSDEFTLKKNILATFA• (359)	*NbPDI16*
C9DFB7	Protein disulfide isomerase OS=*Nicotiana benthamiana*	0.77	0.71	1.45	1.22	Niben101Scf10505g00007	Yes	…EFIEKNRDKPVQSDSARTDSAKDEL• (509)	*NbPDI7*
M0ZSL7	Protein disulfide‐isomerase like OS=*Solanum tuberosum*	0.40	0.26	1.30	1.08	Niben101Scf00332g04004	No	…MLAKSISPAKSDEFTLKKNILATFA• (359)	*NbPDI17*
Q03685	ER luminal‐binding protein 5 OS=*Nicotiana tabacum*	1.49	1.58	1.72	1.49	Niben101Scf08590g00005	Yes	…YQRSGGAPGGASEESNEDDDSHDEL• (668)	*NbBiP1a*
K4C5Z1	ER luminal binding protein OS=*Solanum lycopersicum*	0.98	0.96	1.65	1.42	Niben101Scf08590g00005	Yes	…YQRSGGAPGGASEESNEDDDSHDEL• (668)	*NbBiP1a*
B7U9Z3	ER luminal‐binding protein OS=*Nicotiana benthamiana*	0.94	0.91	1.40	1.18	Niben101Scf02755g06016	Yes	…AVYQKSGGAPGGESGASEDDDHDEL• (655)	*NbBiP1b*
M1AWJ2	ER luminal‐binding protein OS=*Solanum tuberosum*	0.81	0.76	1.32	1.09	Niben101Scf02972g05008	Yes	…TAVYQRSGGAPSGSSAEEEDGHDEL• (666)	*NbBiP2a*
G9MD87	Heat shock protein 90 OS=*Nicotiana tabacum*	1.92	2.11	2.16	1.92	Niben101Scf04331g09018	Yes	…IEEPEAETDEKEAAAKDDSDAKDEL• (811)	*NbGRP94*
M1A384	Calreticulin‐3‐like protein OS=*Solanum tuberosum*	1.48	1.57	1.79	1.56	Niben101Scf10834g03005	Yes	…GRDRYRDRYKKRYHHDYMDDYHDEL• (426)	*NbCRT3*
Q40401	Calreticulin OS=*Nicotiana plumbaginifolia*	1.13	1.14	1.52	1.29	Niben101Scf00332g06011	Yes	…AEEDDDSDDADDKSESKDDEAHDEL• (412)	*NbCRT2*
Q40567	Tobacco calreticulin OS=*Nicotiana tabacum*	0.63	0.54	1.11	0.89	Niben101Scf00466g04036	Yes	…DDADDDSDDADENSESKDDAAHDEL• (416)	*NbCRT1*

Using iTRAQ labelling, changes in protein abundance were evaluated in mock‐infiltrated leaves, leaves expressing P19 only, or leaves co‐expressing P19 and the HA protein from pandemic influenza virus strain H5 Indonesia (H5/A/Indonesia/05/2005; Hamel *et al*., 2023). Using mock samples as a control, pairwise comparisons were performed for P19 and H5 samples. From the resulting lists of up‐regulated proteins, UPR proteins were identified. For all conditions, sampling was performed at 6 DPI. To be considered significantly up‐regulated, proteins had to fulfil the following criterium for at least one of the pairwise comparisons: Log2FC ≥ 1. For each protein retained, UniProt number, annotation, Log2FC value compared to the mock treatment, and deduced *Z*‐score are indicated. Log2FC values that do not meet the minimum threshold are highlighted in red. Niben number of the best corresponding gene is provided, as is the presence or absence of an ER retention signal. For each protein, sequences of the last 25 amino acids are shown, including ER retention signals highlighted in blue. Stop codons are depicted by a black dot (•) and total predicted number of amino acids is shown in parenthesis. On the right, names attributed to each UPR gene is shown.

Our previous transcriptomics and proteomics investigation at 6 DPI had also revealed up‐regulation of cytosolic heat shock proteins (HSPs), a response seen following expression of P19 only, but not following co‐expression of P19 and H5 (Hamel *et al*., 2023). One noticeable exception to this was the gene model Niben101Scf04331g09018, which encodes a molecular chaperone of the HSP90 family. Unlike cytosolic HSPs mentioned above, the protein product from this gene harbours a C‐terminal ‘KDEL’ motif that suggests ER localization. The protein also showed enhanced accumulation following both P19 expression, and co‐expression of P19 and H5 (Table 1). To the best of our knowledge, this HSP90 has never been formally characterized in *N. benthamiana*, although it is annotated as a putative endoplasmin homologue in the improved genome assembly recently published for this plant species (Ranawaka *et al*., 2023). Protein sequence alignments showed that this particular HSP90 homologue shares high homology to Arabidopsis SHEPHERD (AtSHD; AT4G24190), an ER‐resident chaperone involved in the folding of CLAVATA proteins that regulate meristem growth (Ishiguro *et al*., 2002). Product of the Niben101Scf04331g09018 gene is also closely related to the human protein GRP94 (also known as endoplasmin), a key ERAD component that interacts with OS9 to deliver misfolded proteins to the retrotranslocation complex (Christianson *et al*., 2008; Marzec *et al*., 2012; Seidler *et al*., 2014). Herein termed NbGRP94 (Table 1), over‐accumulation of that ER chaperone further suggests that the UPR was activated during H5 protein secretion. We hypothesized that the UPR contributes to efficient expression of the HA protein, in addition of preventing negative effects of the ER stress perhaps induced during enforced expression of the secreted protein.

### Time course sampling, stress symptoms, and recombinant protein accumulation

To assess whether the accumulation of UPR proteins was dependent on early up‐regulation of the corresponding UPR genes, time course sampling was performed at 1, 3, 5, 7, and 9 DPI. For each time point, leaves from non‐infiltrated (NI) plants, or plants only infiltrated with the resuspension buffer (Mock) were used as controls (absence of treatment and effects of the mechanical stress caused by infiltration without the *Agrobacterium* respectively). As the effects of *Agrobacterium* infiltration without recombinant protein expression was previously shown to largely overlap with the effects of *Agrobacterium*‐mediated expression of P19 only (Hamel *et al*., 2023), sampling was also performed on agroinfiltrated leaves solely expressing the viral suppressor of RNA silencing. As such, combined effects of *Agrobacterium* infiltration and P19 expression served as a control for the expression of products with biopharmaceutical interest. For VLPs, leaves co‐expressing P19 along with the HA protein of pandemic influenza virus strain H5 Indonesia (H5/A/Indonesia/05/2005; H5^Indo^) were employed. Hereafter, this recombinant protein will be referred to as H5 (Hamel *et al*., 2023). Regulation of the UPR was also assessed during co‐expression of P19 along with two monoclonal antibodies (mAbs) termed mAb1 and mAb2. Within plant cells, fully assembled antibodies are produced following secretion of antibody light and heavy chains; however, the expression of these proteins does not result in the formation of VLPs. mAb1 and mAb2 were chimeric antibodies that thus comprised variable regions of murine origin and constant regions of human origin. Both antibodies belonged to the gamma (γ) class and were of the same subclass (IgG1). In both cases, light chains belonged to the kappa (κ) type. In other words, primary sequences from mAb1 and mAb2 were exactly the same within constant regions of the light and heavy chains, while variable regions of the light and heavy chains were different between the two antibodies. In addition to their high proximity at the primary protein sequence level, mAb1 and mAb2 were selected based on (1) the contrasted stress symptoms they induced on the host plants, and (2) their contrasting accumulation levels, either high or low, in leaf tissue following heterologous expression (see below).

**Figure 1:**
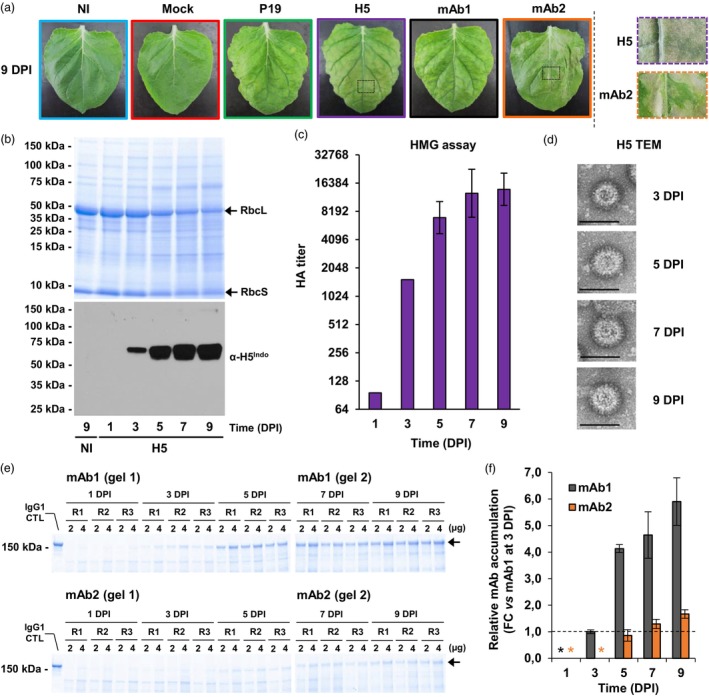
Stress symptoms and recombinant protein accumulation. (a) Stress symptoms observed on representative leaves from each condition harvested 9 days post‐infiltration (DPI). Magnified leaf sections highlight plant cell death on H5 and mAb2 leaves. (b) Total protein extracts from H5 samples following SDS‐PAGE and Coomassie blue staining (upper panel). Arrows highlight RuBisCO small and large subunits (RbcS and RbcL respectively). A Western blot also depicts H5 accumulation at each time point (lower panel). A NI sample harvested at 9 DPI was used as a control lacking H5 expression. (c) For H5 samples, haemagglutination (HMG) assays depict HA protein activity at each time point. (d) For H5 samples, transmission electron microscopy (TEM) confirms production of VLPs at 3, 5, 7, and 9 DPI. Scale bars equal 100 nm. (e) Total protein extracts from mAb1 (top panels) and mAb2 (lower panels) samples after SDS‐PAGE and Coomassie blue staining. Using standard curves made with purified IgG1, gels were used to quantify accumulation of complete monoclonal antibodies (mAb), as measured by densitometry. For each of three repetitions (R1–R3), 2 and 4 μg of total soluble proteins was loaded. Arrows indicate complete IgGs and a purified IgG1 control (CTL) is shown on the left. (f) From densitometry results, fold‐change (FC) accumulation of each mAb was deduced, with accumulation of mAb1 at 3 DPI arbitrarily set at onefold (dashed line). Asterisks denote mAb levels below the limit of quantification, as defined by the standard curves. Condition names are as follows: NI, non‐infiltrated leaves; Mock, leaves infiltrated with buffer only; P19, agroinfiltrated leaves expressing P19 only; H5, agroinfiltrated leaves co‐expressing P19 and H5; mAb1 (mAb2), agroinfiltrated leaves co‐expressing P19 and monoclonal antibody 1 (monoclonal antibody 2).

The effects of foreign protein expression were first characterized by macroscopic evaluation of the stress symptoms induced on representative leaves from each condition harvested at 9 DPI (Figure 1a). Using NI leaves as a baseline, no obvious effect was visible on mock‐infiltrated leaves. For agroinfiltrated leaves expressing P19 only, yellowish discoloration typical of chlorosis was visible, but no sign of plant cell death was denoted. For agroinfiltrated leaves co‐expressing P19 and H5, chlorosis was more advanced and accompanied by diffused greyish necrotic flecking that spread uniformly throughout the leaf blade (see the magnified H5 leaf section; Figure 1a). For agroinfiltrated leaves co‐expressing P19 and mAb1, chlorosis was also observed, but again, no sign of plant cell death was denoted. On the opposite, advanced plant cell death was detected on agroinfiltrated leaves co‐expressing P19 and mAb2. Notably, necrotic lesions found on these leaves were somewhat different from those seen on H5‐expressing leaves, with well‐delimited cell death lesions spanning the entire leaf width (see the magnified mAb2 leaf section; Figure 1a).

To confirm expression of suitable recombinant genes within harvested biomass, real‐time quantitative polymerase chain reaction (RTqPCR) was performed using primers specific to *P19* (Figure S2a), *H5* (Figure S2b), *antibody heavy chain* (*mAb HC*; Figure S2c), and *antibody light chain* (*mAb LC*; Figure S2d). For every gene and time point examined, results confirmed the absence of recombinant gene expression in NI and Mock samples. For every recombinant gene tested, results also indicated that transcript accumulation had not started at 1 DPI. For recombinant gene *P19*, expression was detected in P19, H5, mAb1, and mAb2 samples, with higher expression levels when the *P19* gene was expressed alone. For most conditions, *P19* expression peaked around 3–5 DPI, before going down gradually at later time points (Figure S2a). Consistent with predicted strength of the promoters used to drive recombinant gene expression (*plastocyanin* promoter for *P19* and *2X35S* promoter for other recombinant genes), *P19* was the least expressed when compared to *H5*, *mAb HC*, and *mAb LC* (compare scales from the four panels in Figure S2). For recombinant gene *H5*, expression could only be detected in H5 samples (Figure S2b). *H5* expression rapidly picked up between 1 and 3 DPI, before reaching a peak at 5 DPI. As seen for *P19* (Figure S2a), *H5* expression then gradually decreased at 7 and 9 DPI (Figure S2b). For recombinant genes *mAb HC* (Figure S2c) and *mAb LC* (Figure S2d), expression could only be detected in mAb1 and mAb2 samples. For both genes and for every time point examined, expression in mAb1 samples was higher than in mAb2 samples. During the whole sampling period, both genes displayed overall similar expression profiles; however, *mAb LC* was expressed at much higher levels compared to *mAb HC* (compare scales from Figure S2c,d). Taken together, RTqPCR results confirmed that harvested leaf biomass expressed the proper combinations of recombinant genes, allowing monitoring of recombinant protein accumulation.

To confirm accumulation of the H5 protein, total protein extracts were generated from H5 samples. Sodium dodecyl sulfate–polyacrylamide gel electrophoresis (SDS‐PAGE) followed by Coomassie blue staining confirmed integrity of the proteins from all extracts (upper panel; Figure 1b). As the expression phase progressed, results also revealed gradual reduction in the levels of the Ribulose‐1,5‐bisphosphate carboxylase/oxygenase (RuBisCO), with both RuBisCO subunits similarly affected (upper panel; Figure 1b). This was consistent with what was previously reported when expressing H5 (Hamel *et al*., 2023). From the same protein extracts, a Western blot was then performed using an antibody specific to the HA protein of influenza virus strain H5 Indonesia (α‐H5^Indo^; lower panel; Figure 1b). In H5 samples, no accumulation of the H5 protein was detected at 1 DPI, while low accumulation was seen at 3 DPI. Accumulation of the H5 protein then notably increased between 3 and 5 DPI, and again between 5 and 7 DPI. At that point, H5 accumulation had pretty much reached a plateau, as the level observed at 9 DPI was roughly the same as the one seen at 7 DPI (lower panel; Figure 1b). To assess HA activity, haemagglutination (HMG) assays were conducted on H5 samples (Figure 1c). At 1 DPI, HA activity was barely detectable, consistent with the absence of measurable H5 product (lower panel; Figure 1b). HA activity then notably increased between 1 and 7 DPI, to reach a plateau maintained up to 9 DPI (Figure 1c). Overall, H5 accumulation and HA activity thus correlated tightly, confirming that the recombinant protein H5 accumulated *in planta* was still active against red blood cell receptors following its extraction from the leaf tissues. Using H5 samples harvested at 3, 5, 7, and 9 DPI, VLPs were then partially purified as described previously (D'Aoust et al., 2008). Transmission electron microscopy (TEM) of the purified products confirmed presence of complex structures corresponding to VLPs in both size and morphology, including a lipid membrane covered with spikes that closely resemble those of true influenza virions (Figure 1d).

To monitor the accumulation of recombinant antibodies, total protein extracts were prepared from mAb1 and mAb2 samples. Following SDS‐PAGE under non‐reducing conditions, Coomassie blue staining revealed higher accumulation of mAb1 compared to mAb2, an observation true for every sampling point (Figure 1e). Using stained gels and standard curves made from commercially available preparation of purified IgG1, gel densitometry measurements were next performed on assembled antibodies produced *in planta*. By arbitrarily setting the accumulation of mAb1 at 3 DPI to onefold, relative fold‐change (FC) accumulation of both antibodies was determined at each time point (Figure 1f). For mAb1, no accumulation was detected at 1 DPI, while low but measurable levels were detected at 3 DPI. Levels of the antibody then substantially increased between 3 and 5 DPI, reaching over a fourfold increase compared to the established baseline at 3 DPI. The accumulation rate of mAb1 then decreased between 5 and 9 DPI; yet, the peak of antibody accumulation was not reached until the end of sampling at 9 DPI (Figure 1f). For mAb2, significant product accumulation could not be detected until 5 DPI (Figure 1e,f). The level of mAb2 then slightly increased at 7 and again at 9 DPI, but overall accumulation remained much lower compared to mAb1 (Figure 1e,f). Considering these poor accumulation levels and the strong stress symptoms induced by expression of mAb2 *in planta* (Figure 1a), we hypothesized that this product leads to an unresolved ER stress and therefore that regulation of UPR gene expression within these samples would be informative for comparison with P19, H5, and mAb1 samples.

### Activation of the IRE1‐bZIP60 pathway

**Figure 2:**
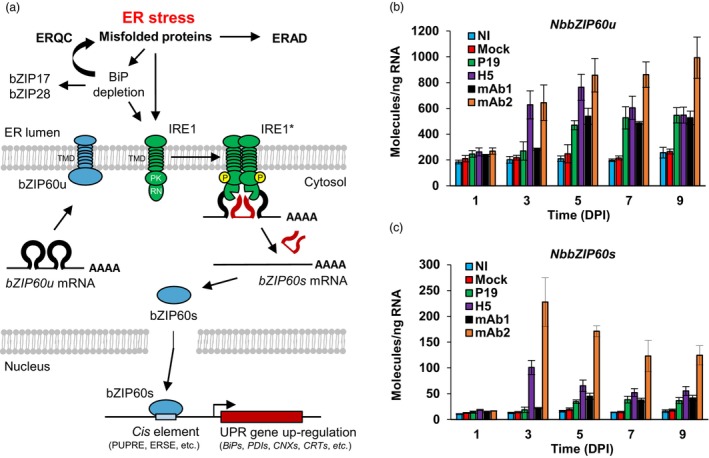
The IRE1‐bZIP60 pathway and up‐regulation of *NbbZIP60*. (a) Model depicting unconventional splicing of *bZIP60* transcripts by IRE1, an ER transmembrane sensor with protein kinase (PK) and ribonuclease (RN) activity. In the absence of stress, unspliced *bZIP60* transcripts (*bZIP60u*) encode a transcription factor (TF) with a transmembrane domain (TMD). Membrane tethering of bZIP60 prevents translocation to the nucleus. ER stress results in misfolded protein accumulation and consequent ERQC and ERAD activation. This triggers IRE1 activation (IRE1*) through oligomerization and phosphorylation (P). Unconventional splicing of *bZIP60* transcripts (*bZIP60s*) results in translation of a shorter TF that lacks a TMD and that can thus induce UPR gene expression in the nucleus. Within the promoter of UPR genes, bZIP60s recognizes *cis* regulatory elements such as the plant unfolded protein response element (UPRE) or the ER stress‐response element (ERSE). Adapted from Duwi Fanata *et al*., 2013. To assess activation of the UPR, expression of *NbbZIP60u* (b) and *NbbZIP60s* (c) was measured by RTqPCR. For each time point in days post‐infiltration (DPI), results are expressed in numbers of molecules per nanogram of RNA. Condition names are as follows: NI, non‐infiltrated leaves; Mock, leaves infiltrated with buffer only; P19, agroinfiltrated leaves expressing P19 only; H5, agroinfiltrated leaves co‐expressing P19 and H5; mAb1 (mAb2), agroinfiltrated leaves co‐expressing P19 and monoclonal antibody 1 (monoclonal antibody 2).

In the absence of ER stress, IRE1 is held in a monomeric, non‐signalling state through interaction with luminal BiPs (Figure 2a). When misfolded proteins start to accumulate, BiPs get recruited for protein folding, freeing the luminal domain of IRE1 for interaction with misfolded proteins that are accumulating. Following oligomerization and phosphorylation, IRE1 gets activated and using its cytosolic RNase domain, it performs unconventional splicing of *bZIP60* transcripts. The shorter TF that is produced lacks a TMD, and as a result, it translocates to the nucleus to up‐regulate expression of UPR genes that carry specific *cis* regulatory elements in their promoter (Figure 2a; Liu and Howell, 2016; Li and Howell, 2021).

To investigate IRE1 activation and unconventional splicing of *bZIP60* transcripts, we retrieved nucleotide sequences of Niben101Scf24096g00018, the gene model encoding *N. benthamiana*'s version of the UPR‐related TF bZIP60. Herein termed *NbbZIP60*, this gene displayed strict sequence conservation around predicted splicing sites recognized by IRE1 (Figure S3). Based on this sequence conservation, forward primers specific to the spliced (s) or unspliced (u) versions of *NbbZIP60* transcripts were designed (Figure S3). Paired to a reverse primer that recognized both transcript versions, forward primers were then used to selectively profile expression of *NbbZIP60u* and *NbbZIP60s* via RTqPCR. For *NbbZIP60u*, results showed similar expression levels for all conditions at 1 DPI (Figure 2b). For NI and Mock samples, expression levels of *NbbZIP60u* remained low and unchanged up to 9 DPI. For H5 and mAb2 samples, up‐regulation of *NbbZIP60u* was seen at 3 DPI and extent of the response was similar for both conditions. In H5 samples, expression of *NbbZIP60u* slightly increased between 3 and 5 DPI, before going down slightly at 7 and 9 DPI. For mAb2 samples, expression level of *NbbZIP60u* also increased between 3 and 5 DPI, but expression levels then remained high up to the end of sampling at 9 DPI. Compared to NI and Mock controls, up‐regulation of *NbbZIP60u* was also observed in P19 and mAb1 samples, but this response arose later (5 DPI) and remained weaker compared to H5 and mAb2 samples (Figure 2b).

For *NbbZIP60s*, overall expression levels were lower than those observed for *NbbZIP60u* (compare scales from Figure 2b,c). This suggests that only a fraction of overall *NbbZIP60* transcripts were unconventionally spliced by IRE1. At 1 DPI, very few spliced transcripts were detected, regardless of the conditions (Figure 2c). Significantly higher levels of *NbbZIP60s* transcripts were however detected for H5 and mAb2 samples at 3 DPI, with significantly higher levels for the latter. For both conditions, levels of spliced transcripts then slowly decreased between 3 and 9 DPI, with levels from mAb2 samples again remaining significantly higher compared to other conditions, including H5 samples. At 5 DPI, H5 samples still displayed significantly higher levels of spliced transcripts compared to P19 samples, but this was no longer the case at 7 and 9 DPI (Figure 2c). For NI and mock samples, levels of spliced transcripts remained low and unchanged throughout the time course. Overall, our data suggest early and transient activation of IRE1 in H5 samples, while early and more sustained activation of IRE1 was seen in mAb2 samples. Compared to NI and mock controls, IRE1 splicing activity was also detected in P19 and mAb1 samples, although arising later and remaining weaker compared to H5 or mAb2 samples.

### Up‐regulation of ERQC genes

**Figure 3:**
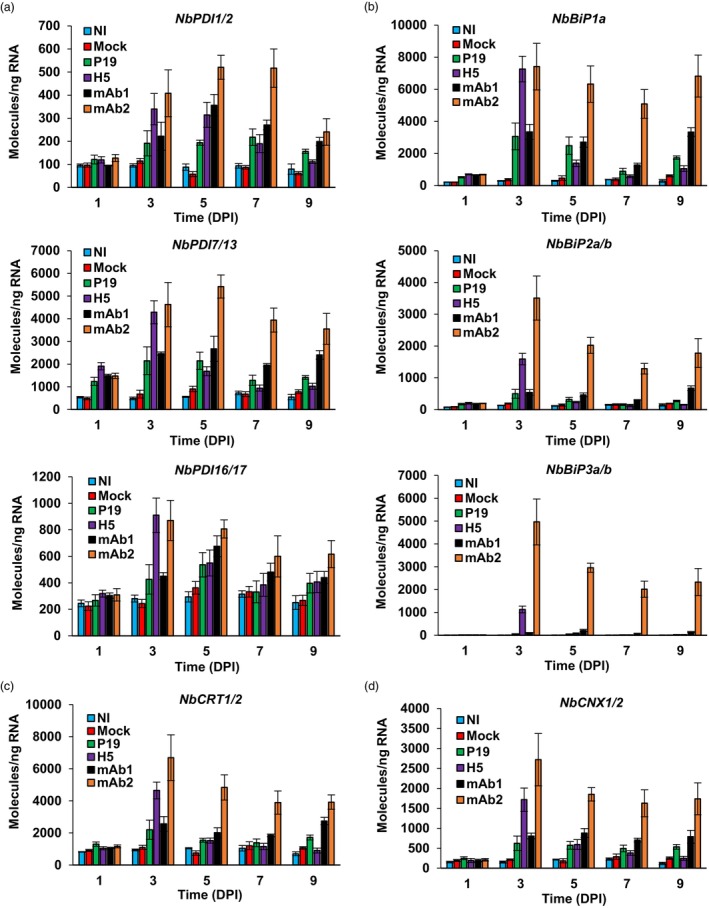
Expression of ERQC genes: *PDIs* and ER‐resident chaperones. Expression of genes encoding PDIs (a) or ER resident chaperones of the BiP (b), CRT (c), and CNX (d) families, as measured by RTqPCR. For each time point in days post‐infiltration (DPI), results are expressed in numbers of molecules per nanogram of RNA. Condition names are as follows: NI, non‐infiltrated leaves; Mock, leaves infiltrated with buffer only; P19, agroinfiltrated leaves expressing P19 only; H5, agroinfiltrated leaves co‐expressing P19 and H5; mAb1 (mAb2), agroinfiltrated leaves co‐expressing P19 and monoclonal antibody 1 (monoclonal antibody 2).

Accumulation of *NbbZIP60s* transcripts (Figure 2c) suggests the UPR to be activated with different strength and kinetics, depending on the foreign protein combinations that were expressed. Activation of the UPR generally leads to the up‐regulation of genes encoding ERQC components, including PDIs and ER‐resident chaperones of the BiP, CRT, and CNX families (Figure 2a; Iwata *et al*., 2008, 2010; Kamauchi *et al*., 2005). Based on proteomics results (Table 1) and homology with homologues from Arabidopsis (Figure S1), primers specific to selected ERQC genes were designed to perform RTqPCR. For NI and mock samples, expression of ERQC genes remained low and essentially unchanged throughout expression (Figure 3a for *NbPDIs*, Figure 3b for *NbBiPs*, Figure 3c for *NbCRTs*, and Figure 3d for *NbCNXs*). For the other conditions, slight differences between respective gene expression profiles were identified; however, three major trends emerged. An early and transient expression pattern was first observed for H5 samples. This pattern was characterized by the up‐regulation of ERQC genes at 3 DPI, followed by expression levels that rapidly decreased to reach levels similar to those observed in P19, mAb1, or even NI and Mock control samples at 5 DPI. The second expression pattern was observed in mAb2 samples and it was also characterized by the early up‐regulation of ERQC genes at 3 DPI. Unlike the first pattern, ERQC gene expression was however sustained at later time points, with levels that never dropped back to basal level and remained significantly higher compared to the other conditions. The third expression pattern, which was observed in P19 and mAb1 samples, was characterized by weaker up‐regulation of ERQC genes at 3 DPI. At later time points, the expression of ERQC genes was above the expression levels observed for NI and mock controls, however expression always remained significantly lower compared to H5 and mAb2 samples. Overall, these observations suggest that at 3 DPI, the UPR was more strongly activated in H5 and mAb2 samples compared to other conditions. For some ERQC genes, they also suggest a higher response level in mAb2 samples compared to H5 samples (e.g. *NbBiP2a/b* and *NbBiP3a/b*; Figure 3b). Expression patterns from all of the ERQC genes examined also tightly correlated with accumulation patterns of *NbbZIP60s* (Figure 2c), suggesting that these UPR genes are direct genetic targets of the TF.

### Up‐regulation of ERAD genes

**Figure 4:**
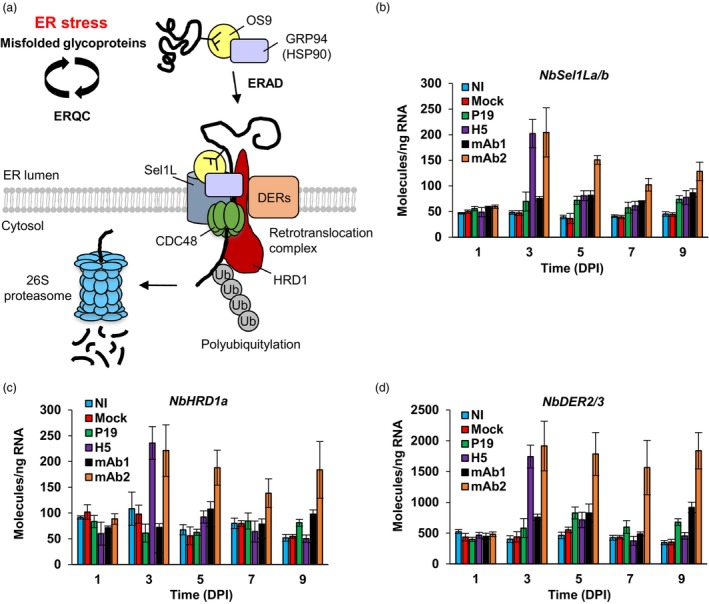
The ERAD system and expression of ERAD genes. (a) Model depicting ER retrotranslocation and cytosolic degradation of a soluble glycoprotein that is irrevocably misfolded. After trimming of its N‐glycan chains, the glycoprotein is recognized by luminal lectin OS9. Together with GRP94, a molecular chaperone of the HSP90 family, OS9 brings the misfolded protein to the retrotranslocation complex. The latter comprises ER membrane proteins Sel1L, HRD1, and Derlins (DERs). Through ATPase motor CDC48, the misfolded glycoprotein is extracted from the ER lumen and released in the cytosol. E3 ubiquitin ligase activity of HRD1 then conjugates ubiquitin (Ub) moieties to the misfolded protein, targeting it for degradation via the 26S proteasome. Adapted from Howell, 2021. Expression of genes encoding Sel1L (b), HRD1 (c), or DER (d) proteins, as measured by RTqPCR. For each time point in days post‐infiltration (DPI), results are expressed in numbers of molecules per nanogram of RNA. Condition names are as follows: NI, non‐infiltrated leaves; Mock, leaves infiltrated with buffer only; P19, agroinfiltrated leaves expressing P19 only; H5, agroinfiltrated leaves co‐expressing P19 and H5; mAb1 (mAb2), agroinfiltrated leaves co‐expressing P19 and monoclonal antibody 1 (monoclonal antibody 2).

In Arabidopsis, activation of the UPR also promotes the expression of ERAD genes (Iwata *et al*., 2008, 2010; Kamauchi *et al*., 2005). These include *Sel1L*, *HRD1*, *DER2*, and *DER3*, which all encode components of the retrotranslocation complex (Figure 4a). To study ERAD gene expression during foreign protein accumulation, we searched the *N. benthamiana* genome to identify ERAD gene homologues. Expression from some of the retrieved candidates was then profiled using RTqPCR. Selected genes were *NbSel1La* and *NbSel1Lb* (Figure 4b), *NbHRD1a* (Figure 4c), as well as the closely related *NbDER2* and *NbDER3* (Figure 4d). At 1 DPI, respective expression levels from all of these genes were similar in all conditions. At 3 DPI, ERAD gene expression significantly increased to similar levels in H5 and mAb2 samples, while P19 and mAb1 samples had similar expression levels compared to NI and Mock controls. At 5 DPI, ERAD gene expression from H5 samples had decreased importantly to reach levels that were similar to P19 and mAb1 samples. After 3 DPI, ERAD gene expression from mAb2 samples also tended to decrease, but it always remained significantly higher compared to the other conditions. Overall expression patterns from the ERAD genes examined were somewhat similar to those seen previously for *NbbZIP60s* (Figure 2c) and ERQC genes (Figure 3).

### Other UPR genes: Subunits of the Sec61 translocon and Bax inhibitor‐1

Translocons are protein complexes that transport nascent proteins from their translation site in the cytosol to the ER lumen. These structures comprise several subunits, including the heterotrimeric complex Sec61, which forms the translocon pore and is made from assembly of secretory (Sec) proteins Sec61α, Sec61β, and Sec61γ (Itskanov and Park, 2023). As a mean to favour protein secretion under stress conditions, genes encoding Sec proteins were found to be induced during the UPR (Iwata *et al*., 2008, 2010; Kamauchi *et al*., 2005). Search of the *N. benthamiana* genome identified three *Sec61α* genes, six *Sec61β* genes, and five almost identical *Sec61γ* genes (Figure S4a). To examine expression of some of these candidates, RTqPCR primers were designed to target *NbSec61α‐1* (Figure S4b), as well as closely related *NbSec61β‐1* and *NbSec61β‐2* (Figure S4c). Similar to ERQC and ERAD genes, results showed enhanced transcript accumulation that started at 3 DPI, a response more importantly induced in H5 and mAb2 samples compared to P19 and mAb1 samples. For mAb2 samples, *NbSec61* gene expression then remained high until the end of sampling at 9 DPI. For H5 samples, expression of these genes on the other hand rapidly decreased between 3 and 5 DPI, reaching levels similar to those observed in P19 and mAb1 samples. No gene induction was seen in NI and mock control samples.

Genes of the *Bax inhibitor‐1* (*BI‐1*) family encode ER transmembrane proteins first identified as suppressors of the cell death induced by pro‐apoptotic protein Bax from yeast and mammals (Hückelhoven, 2004). In severe ER stress cases, BI‐1 protein levels are thought to act as some sort of a rheostat controlling activation of programmed cell death, including in plants (Ishikawa *et al*., 2011). This function perhaps explains why *BI‐1* genes are also induced during the UPR, including in plants (Kamauchi *et al*., 2005; Iwata *et al*., 2008, 2010). Search of the *N. benthamiana* genome identified at least four BI‐1 homologues (NbBI‐1 s), which were most closely related to AtBI‐1a of Arabidopsis (AT5G47120; Figure S4d). Primers specific to *NbBI‐1a* and *NbBI‐1b* showed these genes to be induced at 3 DPI in all agroinfiltrated conditions compared to NI and mock controls. However, up‐regulation levels were significantly higher in H5 and mAb2 samples compared to P19 and mAb1 samples (Figure S4e). At later time points, higher expression seen in H5 samples reverted to levels similar to those seen in P19 and mAb1 samples, while expression remained significantly higher in mAb2 samples. Overall, these results confirmed that the expression of UPR genes of varied functions was induced in H5 and mAb2 samples, and again that these expression profiles mirrored the expression profile of *NbbZIP60s* (Figure 2c).

### Kinetics of defence gene expression versus activation of the UPR

**Figure 5:**
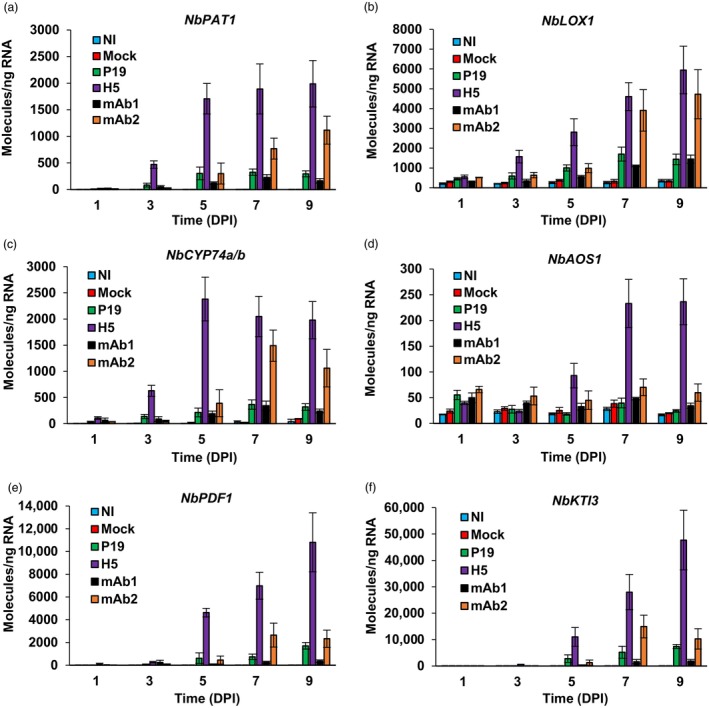
Expression of oxylipin‐related genes. Expression of oxylipin regulatory genes *NbPAT1* (a), *NbLOX1* (b), *NbCYP74a* and *NbCYP74b* (c), as well as *NbAOS1* (d), as measured by RTqPCR. The expression of oxylipin response genes *NbPDF1* (e) and *NbKTI3* (f) was also assessed. These genes were selected because of their responsiveness to H5 protein expression (Hamel *et al*., 2023). For each time point in days post‐infiltration (DPI), results are expressed in numbers of molecules per nanogram of RNA. Condition names are as follows: NI, non‐infiltrated leaves; Mock, leaves infiltrated with buffer only; P19, agroinfiltrated leaves expressing P19 only; H5, agroinfiltrated leaves co‐expressing P19 and H5; mAb1 (mAb2), agroinfiltrated leaves co‐expressing P19 and monoclonal antibody 1 (monoclonal antibody 2).

At 6 DPI, H5 protein expression was shown to induce a number of plant immune responses, including the up‐regulation of oxylipin regulatory and response genes (Hamel *et al*., 2023). As these observations were made at a single time point, we here used RTqPCR to profile expression from some of these defence genes and compared regulation of immune responses with activation timeline of the UPR. For oxylipin regulatory genes, we monitored expression of the patatin gene *NbPAT1*, of the 9‐lipoxygenase gene *NbLOX1*, of divinyl ether synthase (DVE), or epoxy alcohol synthase (EAS) genes *NbCYP74a* and *NbCYP74b*, and of the allene oxide synthase gene *NbAOS1* (Hamel *et al*., 2023). For *NbPAT1* (Figure 5a), *NbLOX1* (Figure 5b), and *NbCYP74* genes (Figure 5c), enhanced gene expression was mostly seen in H5 and mAb2 samples, with higher expression levels detected in the former case. Up‐regulation of these genes also arose faster in H5 samples, with significantly enhanced expression detected at 3 DPI. To a much lesser extent, up‐regulation of these genes was also detected in P19 and mAb1 samples compared to NI and mock controls. This was consistent with the reported low induction of these genes by *Agrobacterium* (Hamel *et al*., 2023). For *NbAOS1*, weak gene up‐regulation was detected in mAb2 samples at 7 and 9 DPI (Figure 5d). Again, gene induction however started earlier (5 DPI) and reached much higher levels in H5 samples.

For oxylipin response genes, expression patterns of the plant defensin gene *NbPDF1* (Figure 5e) and of the Kunitz trypsin inhibitor gene *NbKTI3* (Figure 5f) were examined. As the candidates selected above, these defence genes were previously shown to be highly responsive to H5 protein expression (Hamel *et al*., 2023). Consistent with the induction of oxylipin regulatory genes, RTqPCR revealed strongest up‐regulation in H5 samples. In both cases, enhanced expression was not detected at 3 DPI, but was obvious at 5 DPI (Figure 5e,f). Expression levels from both genes then continued to increase until the end of sampling at 9 DPI. At 7 and 9 DPI, the two genes were also up‐regulated in P19 and mAb2 samples, but expression levels were significantly lower compared to H5 samples. Together, these results indicate that up‐regulation of oxylipin regulatory genes preceded up‐regulation of oxylipin response genes. For H5 samples, these results also indicate that activation of the oxylipin pathway occurred after activation of the UPR, which in this condition peaked at 3 DPI.

**Figure 6:**
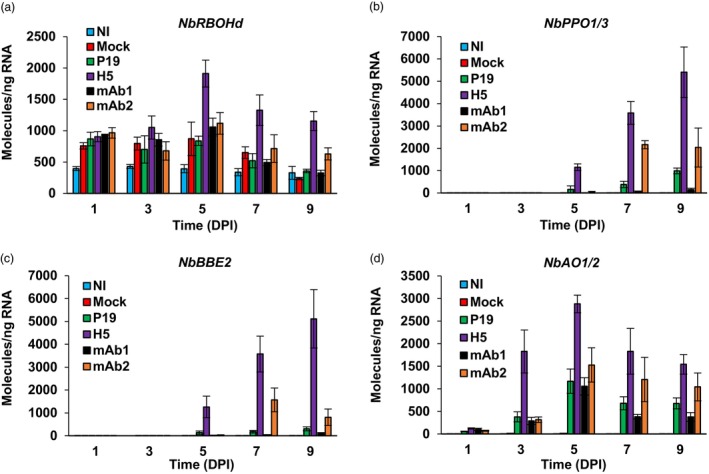
Expression of oxidative stress‐related genes. Expression of *NbRBOHd* (a), *NbPPO1* and *NbPPO3* (b), *NbBBE2* (c), as well as *NbAO1* and *NbAO2* (d), as measured by RTqPCR. These genes were selected because of their responsiveness to H5 protein expression (Hamel *et al*., 2023). For each time point in days post‐infiltration (DPI), results are expressed in numbers of molecules per nanogram of RNA. Condition names are as follows: NI, non‐infiltrated leaves; Mock, leaves infiltrated with buffer only; P19, agroinfiltrated leaves expressing P19 only; H5, agroinfiltrated leaves co‐expressing P19 and H5; mAb1 (mAb2), agroinfiltrated leaves co‐expressing P19 and monoclonal antibody 1 (monoclonal antibody 2).

At 6 DPI, transcriptomics analyses had also shown strong, and in many cases, H5‐specific induction of genes promoting the activation of oxidative stress, including in the apoplast where VLP accumulation takes place (Hamel *et al*., 2023). In view of this, we profiled the expression from some of these genes, namely the NADPH oxidase gene *NbRBOHd*, closely related polyphenol oxidase genes *NbPPO1* and *NbPPO3*, secreted carbohydrate oxidase gene *NbBBE2*, as well as secreted ascorbate oxidase genes *NbAO1* and *NbAO2* (Hamel *et al*., 2023). At 1 DPI, results indicated a similar up‐regulation of *NbRBOHd* in mock and agroinfiltrated samples compared to NI samples (Figure 6a). A comparable expression pattern was observed at 3 DPI but not at 5 DPI, as *NbRBOHd* was expressed at significantly higher level in H5 samples compared to the other agroinfiltrated conditions. In H5 samples, expression of *NbRBOHd* then decreased at 7 and 9 DPI, yet it remained significantly higher compared to P19, mAb1, or mAb2 samples. For *NbPPO* genes (Figure 6b) and *NbBBE2* (Figure 6c), RTqPCR revealed similar expression profiles, with stronger up‐regulation again seen in H5 samples. In the latter samples, up‐regulation was first detected at 5 DPI and then it steadily increased until the end of sampling at 9 DPI. For the last two time points, enhanced gene expression was also detected in mAb2 samples and, to a lower extent, in P19 samples. In both cases, defence gene expression however remained significantly lower compared to H5 samples. No or very limited expression of these genes was detected in NI, mock, or mAb1 samples. For *NbAO* genes (Figure 6d), significant gene induction was detected in all agroinfiltrated leaf samples compared to NI and mock controls. In all cases, the highest up‐regulation level was reached at 5 DPI, even though gene induction started earlier and reached significantly higher levels in H5 samples. Taken together, these results suggest that oxidative stress signalling was stronger in H5 and mAb2 samples, correlating with cell death symptoms observed on leaves expressing these products (Figure 1a). For H5‐expressing samples, up‐regulation of oxidative stress‐related genes again came after the peak of UPR activation at 3 DPI.

## Discussion

Plant molecular farming allows for the large‐scale production of many clinically useful proteins, including therapeutic antibodies and virus surface proteins such as influenza HAs. Plant biotechnology offers alternative approaches to prevent the spreading of infectious diseases and to limit the societal and economic burdens associated with these diseases (Ortiz de Lejarazu‐Leonardo *et al*., 2021). Despite promising developments, mass infiltration of *Agrobacterium* and high yield production of foreign proteins unavoidably put pressure on host plants, including on the cellular machinery mediating protein folding and maturation within the ER. Understanding the impact of those stresses is an important step to ensure sustainability of molecular farming approaches, especially since proteins with biopharmaceutical interest generally require refined protein domain folding and post‐translational modifications such as sophisticated glycosylation patterns or assembly in precise quaternary structures. Overloading the host cell ER with such complex proteins can lead to ER stress and, in turn, in activation of the UPR. When unresolved, ER stress can dramatically compromise plant cell fitness and viability, resulting in poor biomass quality and insufficient protein yields at harvest.

### Protein‐specific activation of the UPR

At 6 DPI, iTRAQ proteomics revealed that *Agrobacterium*‐mediated expression of influenza protein H5 induces accumulation of UPR proteins with varied functions (Table 1; Hamel *et al*., 2023). Interestingly, transcriptomics at 6 DPI did not reveal up‐regulation of the corresponding UPR genes, suggesting this response to be independent of UPR gene induction, or to involve early and transient up‐regulation of these genes prior to biomass harvesting. Here, time course sampling showed that H5 protein expression does in fact result in the induction of UPR genes, a response initiated early after agroinfiltration, that is between 1 and 3 DPI. UPR gene up‐regulation also correlated tightly with unconventional splicing of *NbbZIP60* transcripts, the closest homologue of *AtbZIP60* that largely controls activation of the UPR in Arabidopsis (Iwata *et al*., 2008; Iwata and Koizumi, 2005). Since unconventional splicing of *bZIP60* transcripts reflects the activation status of IRE1, this process is a reliable marker of UPR activation. Consistent with the lack of UPR gene induction at 6 DPI (Hamel *et al*., 2023), H5‐mediated activation of the UPR was transient and could no longer be detected after 5 days of the heterologous protein expression.

In our attempt to better define importance of the UPR during foreign protein expression in *N. benthamiana*, time course sampling of mAb1‐ and mAb2‐expressing samples was also very informative. Despite high similarity between the two IgG1 products, expression profiles of plant genes revealed completely different activation patterns of the UPR. Whereas mAb2 expression induced early, strong, and sustained activation of the UPR, expression of mAb1 resulted in late and much weaker activation of this pathway, with measured levels comparable to those induced by the expression of P19 only. Consistent with this, mAb2 resulted in heavy necrotic symptoms and poor yields, while the mAb1 had little impact on biomass quality despite high product accumulation (Figure 1). Taken together, these results suggest that expression of mAb2 led to an unresolved ER stress, perhaps induced by improper folding of its variable regions, or unsuccessful quaternary protein structure assembly of its light and heavy chains. In any case, these contrasted outcomes highlight importance of the UPR molecular components in guaranteeing success and sustainability of plant molecular farming approaches.

Considering contrasted UPR activation patterns seen for the related mAb1 and mAb2 products, we conclude that H5 protein expression results in moderate activation of the UPR, at least for the viral strain H5^Indo^ that was employed in this study. While induction of UPR genes was obviously higher in H5 samples compared to P19 or mAb1 samples, it was similar or lower than in mAb2 samples, depending on which UPR gene is considered. As for timing, initiation of the UPR occurred within the same time frame in both H5 and mAb2 samples. With regard to UPR activation, the major difference between these two products therefore lied in duration of the response, which was transient in H5 samples and more sustained in mAb2 samples. When expressing recombinant gene *H5*, activation of the UPR also correlated with the initiation of H5 protein accumulation in the biomass between 1 and 3 DPI (Figure 1b). This strongly suggests that the cellular machinery involved in ER protein folding and maturation started to be mobilized as soon as the production of H5 began. Considering that this protein accumulates to levels that sustain the commercial production of influenza vaccine candidates (D'Aoust *et al*., 2008; Landry *et al*., 2010), and that this process results in somewhat mild stress symptoms on the plants (Figure 1a; Hamel *et al*., 2023), early and transient activation of the UPR was apparently sufficient to cope with increased cellular needs associated to foreign H5 protein secretion and to prevent deleterious effects that would have been caused by an unresolved ER stress. It is also important to keep in mind that despite reduced expression of UPR genes between 3 and 5 DPI in H5 samples, proteomics confirms that the level of several UPR proteins was still enhanced at 6 DPI (Table 1; Hamel *et al*., 2023). Cellular benefits from a transient activation of the UPR thus appear to last for a longer time frame, at least during expression of the foreign protein H5.

### Conservation of UPR signalling branches in *N. benthamiana*

For H5 and mAb2 samples, in which activation of the UPR was the strongest, one of the most striking observations is the fact that expression patterns from most UPR genes examined closely mirrored the expression pattern of *NbbZIP60s* (Figure 2c). Since unconventional splicing of *bZIP60* transcripts reflects IRE1 activity, this strongly suggests that this branch of the UPR is involved in foreign protein expression and that UPR genes examined are direct genetic targets of *NbbZIP60*. In plants, the UPR also comprises another key signalling branch, which is initiated by dissociation of BiPs from the ER membrane‐tethered TFs bZIP17 and bZIP28 (Figure 2a; Liu and Howell, 2010; Iwata and Koizumi, 2012; Duwi Fanata *et al*., 2013). These bZIPs can then cruise along the endomembrane system and end up in the Golgi apparatus, where clipping mediated by Golgi‐associated proteases allows for their release from anchoring membrane. Free TFs then translocate to the nucleus, where they up‐regulate the expression of UPR genes in conjunction with bZIP60. Using protein sequences of Arabidopsis bZIP17 and bZIP28 as queries, blast of the *N. benthamiana* genome identified three gene‐encoding proteins that share about 50% identify with their respective homologues in Arabidopsis (Figure S5a). Although low overall, sequence identity of the protein products from Niben101Scf32851g00038 (herein termed *NbbZIP17*), Niben101Scf03647g01004 (*NbbZIP28a*), and Niben101Scf00077g08013 (*NbbZIP28b*) was much higher within functional regions, including the bZIP domain, the TMD, and clipping sites targeted by Golgi‐associated proteases (Figure S5b). Since the promoter region of every UPR gene examined here contains at least one *cis* regulatory element involved in plant UPR signalling (Figure S6), these TFs likely function in conjugation with NbbZIP60 to promote the expression of UPR genes during foreign protein expression. Development of an assay to monitor NbbZIP17 and NbbZIP28 clipping would however be required to formally confirm this hypothesis.

### Activation of the UPR and plant defence gene expression

Considering that expression of influenza H5 induces transient activation of the UPR, we also hypothesize that necrotic symptoms observed on leaves expressing this product are more likely caused by later activation of plant immunity. At 6 DPI, H5 expression was shown to induce a unique molecular signature, including strong and specific up‐regulation of genes involved in oxylipin signalling and responses, as well as in the activation of oxidative stress responses (Hamel *et al*., 2023). As many of these genes typically respond to wounding and herbivory, their up‐regulation is likely associated with budding of the VLPs, which hijack lipids from the PM of plant cells. At the molecular level, these numerous membrane budding events may be perceived as ‘micro‐wounds’ that induce specific plant immunity pathways. While this portrait was drawn using biomass harvested at a single time point after 6 days of expression, the time course approach employed here confirmed that up‐regulation of these defence genes comes after the peak of UPR activation at 3 DPI. Immunoblotting also indicated that defence gene up‐regulation correlated with a high increase in H5 protein accumulation (Figure 1b). Taken as a whole, these findings suggest that early activation of the UPR favours high production of the H5 protein, in turn leading to the activation of plant immunity and eventually in the onset of leaf necrosis. This view is also supported by the fact that spraying of the leaves with an antioxidant solution of ascorbic acid helps to reduce H5‐induced defences and development of leaf necrotic symptoms, with no negative impact on foreign protein accumulation *in planta* (Hamel *et al*., 2023).

In the case of mAb2 samples, in which the strongest necrotic symptoms were induced, expression from most defence genes examined was also induced compared to controls. When compared to H5 samples, induction of these defence genes was however of lower intensity and delayed by several days. As a result, late up‐regulation of these defence genes was perhaps caused by the fact that at this stage of expression, plant cells were already dysfunctional and on the verge of collapsing. On the opposite, expression of UPR genes was induced early at 3 DPI, despite undetectable levels of the mAb2 product until 5 DPI (Figure 1e,f). High UPR gene expression was then sustained up to end of leaf sampling at 9 DPI, suggesting that induced countermeasures never solved the issue associated to the expression of this antibody. In turn, this led to poor accumulation of the recombinant product and to strong activation of plant cell death.

While defence genes that strongly respond to H5 expression were only weakly induced in mAb2 samples, this does not preclude that other defence responses were strongly induced in this condition. In fact, several examples of signalling crosstalk exist between the UPR and plant immunity. In Arabidopsis for instance, the IRE1‐bZIP60 pathway was shown to participate in pathogen responses, with the two IRE1 isoforms having shared and unique functions that can be dependent or independent of bZIP60 (Moreno *et al*., 2012). GTP‐binding protein β1 (AGB1), which is involved in biotic and abiotic stress signalling, was also shown to act synergistically with IRE1 to control activation of the UPR, in addition to immune responses against *Pseudomonas syringae* (Afrin *et al*., 2022). Considering these examples of crosstalk between the UPR and plant immunity, it seems reasonable to think that sustained activation of the UPR in mAb2 samples also led to strong activation of plant immunity in this condition, in turn leading to strong plant cell death activation. While leaves expressing VLP and mAb2 products both displayed necrotic symptoms, the signalling pathways that resulted in plant cell death were most likely different between these two conditions. This perhaps also explains why cell death lesions seen on H5‐expressing leaves were macroscopically different from those seen on leaves expressing mAb2 (Figure 1a).

### Optimization of molecular farming approaches using components of the UPR

Achieving high recombinant protein yields *in planta* is one of the key aspects to ensure success and sustainability of plant molecular farming approaches. While the expression of many foreign proteins is currently performed using wild‐type plants, editing of the host plant genome can be envisioned as a way to optimize productivity or product quality. Alternatively, the co‐expression of helper proteins can lead to an increase in foreign protein yields, as shown in *N. benthamiana* with the use of protease inhibitors that favour the accumulation of recombinant antibodies (Goulet *et al*., 2012; Grosse‐Holz *et al*., 2018; Jutras *et al*., 2016; Robert *et al*., 2013). As for UPR proteins, heterologous expression of a human CRT was shown to improve production of viral glycoproteins that initially showed poor accumulation levels *in planta* (Margolin *et al*., 2020). The results presented here highlight a series of *N. benthamiana* genes that could be used as efficient markers of the UPR activation during stress response or foreign protein expression *in planta*. For complex or even refractory products, the UPR genes here identified may also constitute an interesting list of potential endogenous protein helpers to be co‐expressed as a way to enhance ERQC functions of plant cell biofactories, or to prevent undesirable effects provoked by an unresolved ER stress during foreign protein expression.

## Materials and methods

### Seed germination and plant growth

Seeds of *N. benthamiana* were spread on pre‐wetted peat mix plugs (Ellepot) and placed in a germination chamber for 2 days, where conditions were as follows: 28 °C/28 °C day/night temperature, 16 h photoperiod, 90% relative humidity, and light intensity of 7 μmol m^−2^ s^−1^. Germinated plantlets were next transferred in a growth chamber for 15 days, where conditions were as follows: mean temperature of 28 °C over 24 h, 16 h photoperiod, mean relative humidity of 66% over 24 h, 800 ppm carbon dioxide (CO_2_) injected only during the photo‐phase, and light intensity of 150 μmol m^−2^ s^−1^. During this time, watering and fertilization were provided as needed. After 2 weeks, peat mix plugs were transferred to 4 inches pots containing pre‐wetted peat‐based soil mix (Agro‐Mix). Freshly transferred plantlets were then moved to a greenhouse, where conditions were as follows: mean temperature of 25 °C over 24 h, 16 h photoperiod, mean relative humidity of 66% over 24 h, 800–1000 ppm CO_2_ injected only during the photo‐phase, and light intensity according to natural conditions, but supplemented with artificial high pressure sodium lights at 160 μmol m^−2^ s^−1^. In the greenhouse, watering and fertilization were provided as needed. Growth was allowed to proceed for an average of 20 additional days, until the plants were ready for agroinfiltration.

### Binary vector constructs

For VLP expression, sequences from the mature HA protein of pandemic influenza virus strain H5 Indonesia (H5/A/Indonesia/05/2005; H5^Indo^) were fused to the signal peptide of a *Medicago sativa* (alfalfa) PDI using PCR‐based methods. Once assembled, chimeric *H5* gene was reamplified by PCR and then introduced in the T‐DNA region of a customized pCAMBIA0380 binary vector previously linearized with restriction enzymes *SacII* and *StuI* using the In‐Fusion cloning system (Clontech). The same methods and PDI signal peptide were used to express genes encoding antibody light and heavy chains, which allowed expression of recombinant antibodies mAb1 and mAb2. Expression of recombinant genes *H5*, *mAb LC*, and *mAb HC* was driven by a 2X35S promoter from the cauliflower mosaic virus (CaMV). Expression cassettes also comprised 5′‐ and 3′‐untranslated regions (UTRs) from the cowpea mosaic virus (CPMV), and the *Agrobacterium nopaline synthase* (*NOS*) gene terminator. To prevent silencing induced by recombinant gene expression *in planta*, T‐DNA region of the binary vectors used to express H5 and antibodies also included the suppressor of RNA silencing gene *P19*, under the control of a *plastocyanin* promoter and terminator. For P19 samples, a binary vector allowing expression of P19 only was employed.

### *Agrobacterium* cultures and plant infiltration

Binary vectors were transformed by heat shock in *Agrobacterium* strain AGL1. Transformed bacteria were plated on Luria–Bertani (LB) medium, with appropriate antibiotics selection (kanamycin 50 μg/mL). Colonies were allowed to develop at 28 °C for 2 days. Using isolated colonies, frozen glycerol stocks were prepared and placed at −80 °C for long term storage. When ready, frozen bacterial stocks were thawed at room temperature before transfer in pre‐culture shake flasks containing LB medium with antibiotics selection (kanamycin 50 μg/mL). Bacterial pre‐cultures were grown for 18 h at 28 °C with shaking at 200 rpm. While keeping kanamycin selection, pre‐cultures were transferred to larger shake flasks and bacteria were allowed to develop for an extra 18 h at 28 °C with shaking at 200 rpm. Using a spectrophotometer (Implen), bacterial inoculums were prepared by diluting appropriate volumes of the bacterial cultures in resuspension buffer (10 mM MgCl_2_, 5 mM MES, and pH 5.6). For P19, H5, mAb1, and mAb2 samples, a final OD_600_ of 0.6 was employed for all experiments. Vacuum infiltration was performed by placing whole plant shoots upside down in an airtight stainless‐steel tank containing the appropriate bacterial suspension. To draw air out of the leaves, vacuum pressure was applied for 1 min before pressure release to force the bacterial inoculum into the leaves.

### Transient protein expression and biomass harvesting

Recombinant protein accumulation was allowed to proceed for several days, as indicated. For all experiments, expression took place in condition‐controlled plant growth chambers, where settings were as follows: 20 °C/20 °C day/night temperature, 16 h photoperiod, 80% relative humidity, and light intensity of 150 μmol m^−2^ s^−1^. Watering was performed every other day, with no fertilizer supplied during the expression phase. For biomass harvesting, leaves of similar developmental stage were selected using the leaf plastochron index (Meicenheimer, 2014). The fourth and fifth fully expanded leaves starting from the top of each plant were harvested without petiole. Freshly cut leaves were placed in pre‐frozen 50 mL Falcon tubes, before flash freezing in liquid nitrogen. Frozen biomass was stored at −80 °C until ready for analysis. Using pre‐chilled mortars and pestles, foliar tissue was ground and homogenized into powder using liquid nitrogen. Each sample was made from four leaves collected on two randomly selected plants. The average results presented were obtained from at least three biological replicates.

### Protein extraction and quantification

For protein extraction, 1 g of frozen biomass powder was taken out of the −80 °C freezer and placed on ice. A 2 mL volume of extraction buffer (50 mM Tris, 500 mM NaCl, and pH 8.0) was added, followed by 20 μL of 100 mM phenylmethanesulfonyl fluoride (PMSF) and 2 μL of 0.4 g/mL metabisulfite. Quickly after addition of all solutions, the samples were crushed for 45 s using a Polytron homogenizer (ULTRA‐TURRAX® T25 basic) at maximum speed. One millilitre of each sample was then transferred to a pre‐chilled Eppendorf tube and centrifuged at 10 000 × ***g*** for 10 min at 4 °C. Supernatants were carefully recovered, transferred to new Eppendorf tubes, and kept on ice until determination of protein concentration. To quantify protein content from crude extracts, the Bradford method was employed, with bovine serum albumin as a protein standard.

### Western blotting, HMG assays, and gel densitometry

For Western blotting, total protein extracts were diluted in extraction buffer and mixed with 5X Laemmli sample loading buffer to reach a final concentration of 0.5 μg/μL. Protein samples were denatured at 95 °C for 5 min, followed by a quick spin using a microcentrifuge. Twenty microlitres of each denatured protein extract (10 μg) was then loaded on Criterion™ XT Precast polyacrylamide gels 4%–12% Bis‐Tris and separated at 110 volts for 105 min. Using transfer buffer (25 mM Tris, 192 mM Glycine, and 10% methanol), proteins were next electrotransferred onto a polyvinylidene difluoride (PVDF) membrane at 100 volts. After 30 min, the membranes were placed in blocking solution: 1X Tris‐Buffered Saline with Tween‐20 (TBS‐T; 50 mM Tris, pH 7.5, 150 mM NaCl, and 0.1% (v/v) Tween‐20), with 5% nonfat dried milk. Membranes were blocked overnight at 4 °C with gentle shaking. The next morning, blocking solution was removed and primary antibodies were incubated at room temperature for 60 min with gentle shaking in 1X TBS‐T, 2% nonfat dried milk solution. After four washes in 1X TBS‐T, secondary antibodies were added and incubated at room temperature for 60 min with gentle shaking in 1X TBS‐T, 2% nonfat dried milk solution. After four extra washes in 1X TBS‐T, Luminata™ Western HRP Chemiluminescence Substrate (Thermo Fisher Scientific) was added to the membranes and protein complexes were visualized under the chemiluminescence mode of an Imager 600 apparatus (Amersham). Antibody dilutions were as follows: anti‐HA A/Indonesia/05/2005 (H5N1; CBER): 1/5000 (primary antibody). Rabbit anti‐sheep (JIR): 1/10 000 (secondary antibody).

For HMG assays, turkey red blood cells were diluted to a concentration of 0.25% (v/v) in phosphate‐buffered saline solution (PBS; 0.1 M PO_4_, 0.15 M NaCl, and pH 7.2). While keeping red blood cells on ice, protein samples were diluted in extraction buffer using 1/384 and 1/576 ratios. For each dilution, 200 μL of total protein extract was transferred to the first row of a 96‐well plate. Eight serial dilutions were next performed using 100 μL of protein extract mixed to 100 μL of PBS buffer previously poured in each plate well. Following serial dilutions, 100 μL of the red blood cell solution was added to protein extracts. After thorough mixing, samples were incubated overnight at room temperature. HA activity was scored visually on the next day.

For gel densitometry, total protein extracts were diluted in extraction buffer and mixed with 5X Laemmli sample loading buffer without reducing reagent. Protein samples were then denatured at 95 °C for 5 min, followed by a quick spin using a microcentrifuge. For each sample, 2 and 4 μg of denatured protein extracts were loaded on Criterion™ XT Precast polyacrylamide gels 4%–12% Bis‐Tris. To quantify fully assembled antibodies, a standard curve consisting of 1.5 μg–0.125 μg of purified IgG1 (Sigma) was added on each gel. Protein separation was done at 110 volts for 105 min and staining of the gels was done using Coomassie blue.

### RNA extractions and RNA quantification

Using the RNeasy commercial kit (Qiagen), 100 mg of frozen biomass powder was used for RNA extractions. Residual DNA was removed using the RNase‐free DNase Set (Qiagen). Concentration of RNA extracts was determined using a spectrophotometer (Implen) and integrity evaluated using a 2100 BioAnalyzer (Agilent). For long‐term storage, RNA extracts were stabilized by adding the RNAseOUT recombinant ribonuclease inhibitor (Thermo Fisher Scientific), before freezing at −80 °C until further analysis.

### RTqPCR analyses

For each sample, 1 μg of RNA was reverse transcribed into cDNA using the QuantiTect Reverse Transcription Kit (Qiagen). Transcript quantification was performed in 96‐well plates, using the ABI PRISM 7500 Fast real‐time PCR system and custom data analysis software (Thermo Fisher Scientific). Each reaction contained the equivalent of 5 ng cDNA as a template, 0.5 μM of forward and reverse primers, and 1X QuantiTect SYBR Green Master Mix (Qiagen) for a total reaction volume of 10 μL. RTqPCR runs were done under the SYBR Green amplification mode and cycling conditions were as follows: 15 min incubation at 95 °C, followed by 40 amplification cycles at 95 °C for 5 s, 60 °C for 30 s, and 65 °C for 90 s. Reactions in the absence of cDNA template were conducted as negative controls and melting curve analyses were performed to confirm the lack of primer dimer formation and amplification specificity. Resulting fluorescence and cycle threshold (Ct) values were next exported to the Microsoft Excel software. To correct for biological variability and technical variations during RNA extraction, RNA quantification, or reverse transcription, expression from six housekeeping genes (*NbACT1*, *NbVATP*, *NbSAND*, *NbUBQ1*, *NbEF1‐α*, and *NbGAPDH1*) was used to normalize expression data (Hamel *et al*., 2023; Vandesompele *et al*., 2002). Normalized numbers of transcript molecules per nanogram of RNA were deduced using the 2^−ΔΔCt^ method (Bustin *et al*., 2009; Livak and Schmittgen, 2001) and standard curves derived from known quantities of phage lambda DNA. Standard deviation related to the within‐treatment biological variation was calculated in accordance with the error propagation rules. Sequences of all primers used in this study are available in the Table S2.

### Phylogenetic analyses

For each protein family to be analysed, the genome of *N. benthamiana* (https://solgenomics.net/) was searched using full‐length amino acid sequences of Arabidopsis homologues as queries. Full‐length protein sequences retrieved from blast analyses were next aligned with ClustalW. Alignment parameters were as follows: for pairwise alignments, 10.0 for gap opening and 0.1 for gap extension. Parameters for multiple alignments were 10.0 for gap opening and 0.20 for gap extension. The resulting alignments were submitted to the MEGA5 software and neighbour‐joining trees derived from 5000 replicates were generated. Bootstrap values are indicated on the node of each branch. More details on phylogenetic analyses are provided in the legend of relevant figures.

### Statistical analyses

Statistical analyses on RTqPCR data were performed on Graph Pad Prism 9.5.0, using two‐way ANOVA with time points as row factors and conditions as column factors. Conditions were then analysed by a *post hoc* Tukey's multiple comparison test with an alpha threshold of 0.05. For clarity and to limit the number of statistical groups generated, control conditions (NI and mock) were omitted from statistical analyses, which were thus only performed on agroinfiltrated conditions (P19, H5, mAb1, and mAb2). Groups are labelled with a compact letter display. Groups that do not share the same letter(s) are statistically different. Complete results from statistical analyses can be found in the Table S3.

### Search for *cis* regulatory elements

To locate *cis* regulatory elements within the promoter of UPR genes, the putative promoter regions of each gene tested were retrieved from the genome sequences of *N. benthamiana* (https://solgenomics.net/). For the analysis, a 1000 bp region located upstream of the annotated start codon was employed. Motif search was performed in both sequence orientations using the Find Individual Motif Occurrences (FIMO) software (Grant *et al*., 2011). As they were previously involved in the UPR of plants (Iwata *et al*., 2008; Iwata and Koizumi, 2005; Liu and Howell, 2016), *cis* regulatory elements that were examined were as follows: PUPRE (ATTGGTCCACGTCATC), ERSE (CCAATN_10_CACG), ERSE2 (ATTGGN_2_CACG), UPRE2 (GATGACGCGTAC), UPRE3 (TCATCG), and XBP‐BS (GATGACGTGK).

## Conflicts of interest

At the time of this work, L.P.H., M.A.C., R.T., F.P.G., M.E.P., P.O.L., and M.A.D. were employees of Medicago Inc. M.C.G. and D.M. declare that the research was conducted in the absence of any commercial or financial relationships that could be construed as a potential conflict of interest.

## Author contributions

L.P.H., D.M., and M.A.D. designed the research, supervised the project, and analysed the data. L.P.H. and M.A.C. assembled the figures and drafted the article. P.O.L. managed the production of genetic constructs used to express influenza H5 and antibodies. M.C.G. contributed to the proteomics study. L.P.H., F.P.G., and M.A.C. managed data and performed searches within RNAseq and proteomics data. R.T., F.P.G., M.E.P., and M.A.C. performed RTqPCR, assays for recombinant protein quantification and Western blots. M.E.P. performed VLP purification and TEM imaging. F.P.G. and M.A.C. performed the statistical analyses. M.A.C. performed searches for *cis* regulatory elements. All authors read, helped to edit, and approved the final version of the article.

## Supporting information


**Table S1** Name and identification number of UPR genes from *A. thaliana* and *N. benthamiana*.

**Table S2** RTqPCR primers used in this study.

**Table S3** Statistical analyses of the RTqPCR results.

**Figure S1** Phylogeny of *N. benthamiana* PDIs and ER‐resident chaperones.
**Figure S2** Expression of recombinant genes.
**Figure S3** Primer design to assess unconventional splicing of *NbbZIP60*.
**Figure S4** Phylogeny of other UPR proteins and expression of some of their corresponding genes.
**Figure S5** Phylogeny of UPR activating bZIPs and conservation of bZIP17 and bZIP28 in *N. benthamiana*.
**Figure S6** *Cis* regulatory elements in the promoter of UPR genes.

## Data Availability

All data discussed in this study can be found in the article and in the Supplementary Materials.

## References

[pbi14252-bib-0001] Afrin, T. , Costello, C.N. , Monella, A.N. , Kørner, C.J. and Pajerowska‐Mukhtar, K.M. (2022) The interplay of GTP‐binding protein AGB1 with ER stress sensors IRE1a and IRE1b modulates Arabidopsis unfolded protein response and bacterial immunity. Plant Signal. Behav. 17, e2018857. 10.1080/15592324.2021.2018857 PMC8920210 34968413

[pbi14252-bib-0002] Araki, K. and Nagata, K. (2011) Protein folding and quality control in the ER. Cold Spring Harb. Perspect. Biol. 3, a007526. 21875985 10.1101/cshperspect.a007526 PMC3220362

[pbi14252-bib-0003] Bally, J. , Jung, H. , Mortimer, C. , Naim, F. , Philips, J.G. , Hellens, R. , Bombarely, A. *et al*. (2018) The rise and rise of *Nicotiana benthamiana*: a plant for all reasons. Annu. Rev. Phytopathol. 56, 405–426. 30149789 10.1146/annurev-phyto-080417-050141

[pbi14252-bib-0004] Benham, A.M. (2012) Protein secretion and the endoplasmic reticulum. Cold Spring Harb. Perspect. Biol. 4, a012872. 22700933 10.1101/cshperspect.a012872 PMC3405867

[pbi14252-bib-0005] Brodsky, J.L. and Wojcikiewicz, R.J. (2009) Substrate‐specific mediators of ER associated degradation (ERAD). Curr. Opin. Cell Biol. 21, 516–521. 19443192 10.1016/j.ceb.2009.04.006 PMC2756615

[pbi14252-bib-0006] Bustin, S.A. , Benes, V. , Garson, J.A. , Hellemans, J. , Huggett, J. , Kubista, M. , Mueller, R. *et al*. (2009) The MIQE guidelines: minimum information for publication of quantitative real‐time PCR experiments. Clin. Chem. 55, 611–622. 19246619 10.1373/clinchem.2008.112797

[pbi14252-bib-0007] Christianson, J.C. , Shaler, T.A. , Tyler, R.E. and Kopito, R.R. (2008) OS‐9 and GRP94 deliver mutant α_1_‐antitrypsin to the Hrd1‐SEL1L ubiquitin ligase complex for ERAD. Nat. Cell Biol. 10, 272–282. 18264092 10.1038/ncb1689 PMC2757077

[pbi14252-bib-0008] Chung, Y.H. , Church, D. , Koellhoffer, E.C. , Osota, E. , Shukla, S. , Rybicki, E.P. , Pokorski, J.K. *et al*. (2022) Integrating plant molecular farming and materials research for next‐generation vaccines. Nat. Rev. Mater. 7, 372–388. 34900343 10.1038/s41578-021-00399-5 PMC8647509

[pbi14252-bib-0009] D'Aoust, M.A. , Lavoie, P.O. , Couture, M.M. , Trepanier, S. , Guay, J.M. , Dargis, M. , Mongrand, S. *et al*. (2008) Influenza virus‐like particles produced by transient expression in *Nicotiana benthamiana* induce a protective immune response against a lethal viral challenge in mice. Plant Biotechnol. J. 6, 930–940. 19076615 10.1111/j.1467-7652.2008.00384.x

[pbi14252-bib-0010] Duwi Fanata, W.I. , Lee, S.Y. and Lee, K.O. (2013) The unfolded protein response in plants: a fundamental adaptive cellular response to internal and external stresses. J. Proteomics, 93, 356–368. 23624343 10.1016/j.jprot.2013.04.023

[pbi14252-bib-0011] Goodin, M.M. , Zaitlin, D. , Naidu, R.A. and Lommel, S.A. (2008) *Nicotiana benthamiana*: its history and future as a model for plant‐pathogen interactions. Mol. Plant Microbe Interact. 21, 1015–1026. 18616398 10.1094/MPMI-21-8-1015

[pbi14252-bib-0012] Goulet, C. , Khalf, M. , Sainsbury, F. , D'Aoust, M.A. and Michaud, D. (2012) A protease activity‐depleted environment for heterologous proteins migrating towards the leaf cell apoplast. Plant Biotechnol. J. 10, 83–94. 21895943 10.1111/j.1467-7652.2011.00643.x

[pbi14252-bib-0013] Grant, C.E. , Bailey, T.L. and Noble, W.S. (2011) FIMO: scanning for occurrences of a given motif. Bioinformatics, 27, 1017–1018. 21330290 10.1093/bioinformatics/btr064 PMC3065696

[pbi14252-bib-0014] Grosse‐Holz, F. , Madeira, L. , Zahid, M.A. , Songer, M. , Kourelis, J. , Fesenko, M. , Ninck, S. *et al*. (2018) Three unrelated protease inhibitors enhance accumulation of pharmaceutical recombinant proteins in *Nicotiana benthamiana* . Plant Biotechnol. J. 16, 1797–1810. 29509983 10.1111/pbi.12916 PMC6131417

[pbi14252-bib-0015] Hamel, L.P. , Tardif, R. , Poirier‐Gravel, F. , Rasoolizadeh, A. , Brosseau, C. , Giroux, G. , Lucier, J.F. *et al*. (2023) Molecular responses of agroinfiltrated *Nicotiana benthamiana* leaves expressing suppressor of silencing P19 and influenza virus‐like particles. Plant Biotechnol. 10.1111/pbi.14247 PMC11022802 38041470

[pbi14252-bib-0016] Howell, S.H. (2013) Endoplasmic reticulum stress responses in plants. Annu. Rev. Plant Biol. 64, 477–499. 23330794 10.1146/annurev-arplant-050312-120053

[pbi14252-bib-0017] Howell, S.H. (2021) Evolution of the unfolded protein response in plants. Plant Cell Environ. 44, 2625–2635. 33840122 10.1111/pce.14063

[pbi14252-bib-0018] Hückelhoven, R. (2004) BAX Inhibitor‐1, an ancient cell death suppressor in animals and plants with prokaryotic relatives. Apoptosis, 9, 299–307. 15258461 10.1023/b:appt.0000025806.71000.1c

[pbi14252-bib-0019] Hüttner, S. , Veit, C. , Schoberer, J. , Grass, J. and Strasser, R. (2012) Unraveling the function of *Arabidopsis thaliana* OS9 in the endoplasmic reticulum‐associated degradation of glycoproteins. Plant Mol. Biol. 79, 21–33. 22328055 10.1007/s11103-012-9891-4 PMC3332353

[pbi14252-bib-0020] Ishiguro, S. , Watanabe, Y. , Ito, N. , Nonaka, H. , Takeda, N. , Sakai, T. , Kanaya, H. *et al*. (2002) SHEPHERD is the *Arabidopsis* GRP94 responsible for the formation of functional CLAVATA proteins. EMBO J. 21, 898–908. 11867518 10.1093/emboj/21.5.898 PMC125899

[pbi14252-bib-0021] Ishikawa, T. , Watanabe, N. , Nagano, M. , Kawai‐Yamada, M. and Lam, E. (2011) Bax inhibitor‐1: a highly conserved endoplasmic reticulum‐resident cell death suppressor. Cell Death Differ. 18, 1271–1278. 21597463 10.1038/cdd.2011.59 PMC3172100

[pbi14252-bib-0022] Itskanov, S. and Park, E. (2023) Mechanism of Protein Translocation by the Sec61 Translocon Complex. Cold Spring Harb. Perspect. Biol. 15, a041250. 35940906 10.1101/cshperspect.a041250 PMC9808579

[pbi14252-bib-0023] Iwata, Y. and Koizumi, N. (2005) An *Arabidopsis* transcription factor, AtbZIP60, regulates the endoplasmic reticulum stress response in a manner unique to plants. Proc. Natl. Acad. Sci. U. S. A. 102, 5280–5285. 15781873 10.1073/pnas.0408941102 PMC555978

[pbi14252-bib-0024] Iwata, Y. and Koizumi, N. (2012) Plant transducers of the endoplasmic reticulum unfolded protein response. Trends Plant Sci. 17, 720–727. 22796463 10.1016/j.tplants.2012.06.014

[pbi14252-bib-0025] Iwata, Y. , Fedoroff, N.V. and Koizumi, N. (2008) *Arabidopsis* bZIP60 is a proteolysis‐activated transcription factor involved in the endoplasmic reticulum stress response. Plant Cell, 20, 3107–3121. 19017746 10.1105/tpc.108.061002 PMC2613661

[pbi14252-bib-0026] Iwata, Y. , Sakiyama, M. , Lee, M.H. and Koizumi, N. (2010) Transcriptomic response of *Arabidopsis thaliana* to tunicamycin‐induced endoplasmic reticulum stress. Plant Biotechnol. 27, 161–171.

[pbi14252-bib-0027] Jakoby, M. , Weisshaar, B. , Dröge‐Laser, W. , Vicente‐Carbajosa, J. , Tiedemann, J. , Kroj, T. and Parcy, F. (2002) bZIP transcription factors in *Arabidopsis* . Trends Plant Sci. 7, 106–111. 11906833 10.1016/s1360-1385(01)02223-3

[pbi14252-bib-0028] Jutras, P.V. , Marusic, C. , Lonoce, C. , Deflers, C. , Goulet, M.C. , Benvenuto, E. , Michaud, D. *et al*. (2016) An accessory protease inhibitor to increase the yield and quality of a tumour‐targeting mAb in *Nicotiana benthamiana* leaves. PloS One, 11, e0167086. 27893815 10.1371/journal.pone.0167086 PMC5125672

[pbi14252-bib-0029] Kamauchi, S. , Nakatani, H. , Nakano, C. and Urade, R. (2005) Gene expression in response to endoplasmic reticulum stress in *Arabidopsis thaliana* . FEBS J. 272, 3461–3476. 15978049 10.1111/j.1742-4658.2005.04770.x

[pbi14252-bib-0030] Kørner, C.J. , Du, X. , Vollmer, M.E. and Pajerowska‐Mukhtar, K.M. (2015) Endoplasmic reticulum stress signaling in plant immunity ‐ at the crossroad of life and death. Int. J. Mol. Sci. 16, 26582–26598. 26556351 10.3390/ijms161125964 PMC4661823

[pbi14252-bib-0031] Landry, N. , Ward, B.J. , Trepanier, S. , Montomoli, E. , Dargis, M. , Lapini, G. and Vezina, L.P. (2010) Preclinical and clinical development of plant‐made virus‐like particle vaccine against avian H5N1 influenza. PloS One, 5, e15559. 21203523 10.1371/journal.pone.0015559 PMC3008737

[pbi14252-bib-0032] Li, Z. and Howell, S.H. (2021) Review: The two faces of IRE1 and their role in protecting plants from stress. Plant Sci. 303, 110758. 33487343 10.1016/j.plantsci.2020.110758

[pbi14252-bib-0033] Liu, J.X. and Howell, S.H. (2010) Endoplasmic reticulum protein quality control and its relationship to environmental stress responses in plants. Plant Cell, 22, 2930–2942. 20876830 10.1105/tpc.110.078154 PMC2965551

[pbi14252-bib-0034] Liu, J.X. and Howell, S.H. (2016) Managing the protein folding demands in the endoplasmic reticulum of plants. New Phytol. 211, 418–428. 26990454 10.1111/nph.13915

[pbi14252-bib-0035] Liu, J.X. , Srivastava, R. , Che, P. and Howell, S.H. (2007) An endoplasmic reticulum stress response in *Arabidopsis* is mediated by proteolytic processing and nuclear relocation of a membrane‐associated transcription factor, bZIP28. Plant Cell, 19, 4111–4119. 18156219 10.1105/tpc.106.050021 PMC2217655

[pbi14252-bib-0036] Livak, K.J. and Schmittgen, T.D. (2001) Analysis of relative gene expression data using real‐time quantitative PCR and the 2^−∆∆Ct^ Method. Methods, 25, 402–448. 11846609 10.1006/meth.2001.1262

[pbi14252-bib-0037] Lu, D.P. and Christopher, D.A. (2008) Endoplasmic reticulum stress activates the expression of a sub‐group of protein disulfide isomerase genes and AtbZIP60 modulates the response in *Arabidopsis thaliana* . Mol. Genet. Genomics, 280, 199–210. 18574595 10.1007/s00438-008-0356-z

[pbi14252-bib-0038] Margolin, E. , Oh, Y.J. , Verbeek, M. , Naude, J. , Ponndorf, D. , Meshcheriakova, Y.A. , Peyret, H. *et al*. (2020) Co‐expression of human calreticulin significantly improves the production of HIV gp140 and other viral glycoproteins in plants. Plant Biotechnol. J. 18, 2109–2117. 32096288 10.1111/pbi.13369 PMC7540014

[pbi14252-bib-0039] Marzec, M. , Eletto, D. and Argon, Y. (2012) GRP94: an HSP90‐like protein specialized for protein folding and quality control in the endoplasmic reticulum. Biochim. Biophys. Acta, 1823, 774–787. 22079671 10.1016/j.bbamcr.2011.10.013 PMC3443595

[pbi14252-bib-0040] Meicenheimer, R.D. (2014) The plastochron index: still useful after nearly six decades. Am. J. Bot. 101, 1821–1835. 25366849 10.3732/ajb.1400305

[pbi14252-bib-0041] Moreno, A.A. , Mukhtar, M.S. , Blanco, F. , Boatwright, J.L. , Moreno, I. , Jordan, M.R. , Chen, Y. *et al*. (2012) IRE1/bZIP60‐mediated unfolded protein response plays distinct roles in plant immunity and abiotic stress responses. PloS One, 7, e31944. 22359644 10.1371/journal.pone.0031944 PMC3281089

[pbi14252-bib-0042] Munro, S. and Pelham, H.R. (1987) A C‐terminal signal prevents secretion of luminal ER proteins. Cell, 48, 899–907. 3545499 10.1016/0092-8674(87)90086-9

[pbi14252-bib-0043] Nagashima, Y. , Mishiba, K. , Suzuki, E. , Shimada, Y. , Iwata, Y. and Koizumi, N. (2011) *Arabidopsis* IRE1 catalyses unconventional splicing of *bZIP60* mRNA to produce the active transcription factor. Sci. Rep. 1, 29. 22355548 10.1038/srep00029 PMC3216516

[pbi14252-bib-0044] Ortiz de Lejarazu‐Leonardo, R. , Montomoli, E. , Wojcik, R. , Christopher, S. , Mosnier, A. , Pariani, E. , Trilla Garcia, A. *et al*. (2021) Estimation of reduction in influenza vaccine effectiveness due to egg‐adaptation changes‐systematic literature review and expert consensus. Vaccines (Basel), 9, 1255. 34835186 10.3390/vaccines9111255 PMC8621612

[pbi14252-bib-0045] Ranawaka, B. , An, J. , Lorenc, M.T. , Jung, H. , Sulli, M. , Aprea, G. , Roden, S. *et al*. (2023) A multi‐omic *Nicotiana benthamiana* resource for fundamental research and biotechnology. Nat. Plants, 9, 1558–1571. 37563457 10.1038/s41477-023-01489-8 PMC10505560

[pbi14252-bib-0046] Read, A. and Schröder, M. (2021) The unfolded protein response: an overview. Biology (Basel), 10, 384. 33946669 10.3390/biology10050384 PMC8146082

[pbi14252-bib-0047] Robert, S. , Khalf, M. , Goulet, M.C. , D'Aoust, M.A. , Sainsbury, F. and Michaud, D. (2013) Protection of recombinant mammalian antibodies from development‐dependent proteolysis in leaves of *Nicotiana benthamiana* . PloS One, 8, e70203. 23894618 10.1371/journal.pone.0070203 PMC3720903

[pbi14252-bib-0048] Seidler, P.M. , Shinsky, S.A. , Hong, F. , Li, Z. , Cosgrove, M.S. and Gewirth, D.T. (2014) Characterization of the Grp94/OS‐9 chaperone‐lectin complex. J. Mol. Biol. 426, 3590–3605. 25193139 10.1016/j.jmb.2014.08.024 PMC4188734

[pbi14252-bib-0049] Silhavy, D. , Molnár, A. , Lucioli, A. , Szittya, G. , Hornyik, C. , Tavazza, M. and Burgyán, J. (2002) A viral protein suppresses RNA silencing and binds silencing‐generated, 21‐ to 25‐nucleotide double‐stranded RNAs. EMBO J. 21, 3070–3080. 12065420 10.1093/emboj/cdf312 PMC125389

[pbi14252-bib-0050] Stotz, H.U. , Mueller, S. , Zoeller, M. , Mueller, M.J. and Berger, S. (2013) TGA transcription factors and jasmonate‐independent COI1 signalling regulate specific plant responses to reactive oxylipins. J. Exp. Bot. 64, 963–975. 23349138 10.1093/jxb/ers389 PMC3580818

[pbi14252-bib-0051] Vandesompele, J. , De Preter, K. , Pattyn, F. , Poppe, B. , Van Roy, N. , De Paepe, A. and Speleman, F. (2002) Accurate normalization of real‐time quantitative RT‐PCR data by geometric averaging of multiple internal control genes. Genome Biol. 3(Research0034), 1. 10.1186/gb-2002-3-7-research0034 PMC126239 12184808

[pbi14252-bib-0052] Wu, X. and Rapoport, T.A. (2018) Mechanistic insights into ER‐associated protein degradation. Curr. Opin. Cell Biol. 53, 22–28. 29719269 10.1016/j.ceb.2018.04.004 PMC6131047

