# Supplementary material for: Heterologous expression of influenza haemagglutinin leads to early and transient activation of the unfolded protein response in Nicotiana benthamiana
Source: Plant Biotechnol J. 2023 Dec 1;22(5):1146–63. doi: 10.1111/pbi.14252 (PMC11022800; doi:10.1111/pbi.14252)
Supplement: Supplementary file 4 — Figure S1 Phylogeny of N. benthamiana PDIs and ER‐resident chaperones. Figure S2 Expression of recombinant genes. Figure S3 Primer design to assess unconventional splicing of NbbZIP60. Figure S4 Phylogeny of other UPR proteins and expression of some of their corresponding genes. Figure S5 Phylogeny of UPR activating bZIPs and conservation of bZIP17 and bZIP28 in N. benthamiana. Figure S6 Cis regulatory elements in the promoter of UPR genes. [file PBI-22-1146-s003.pdf]

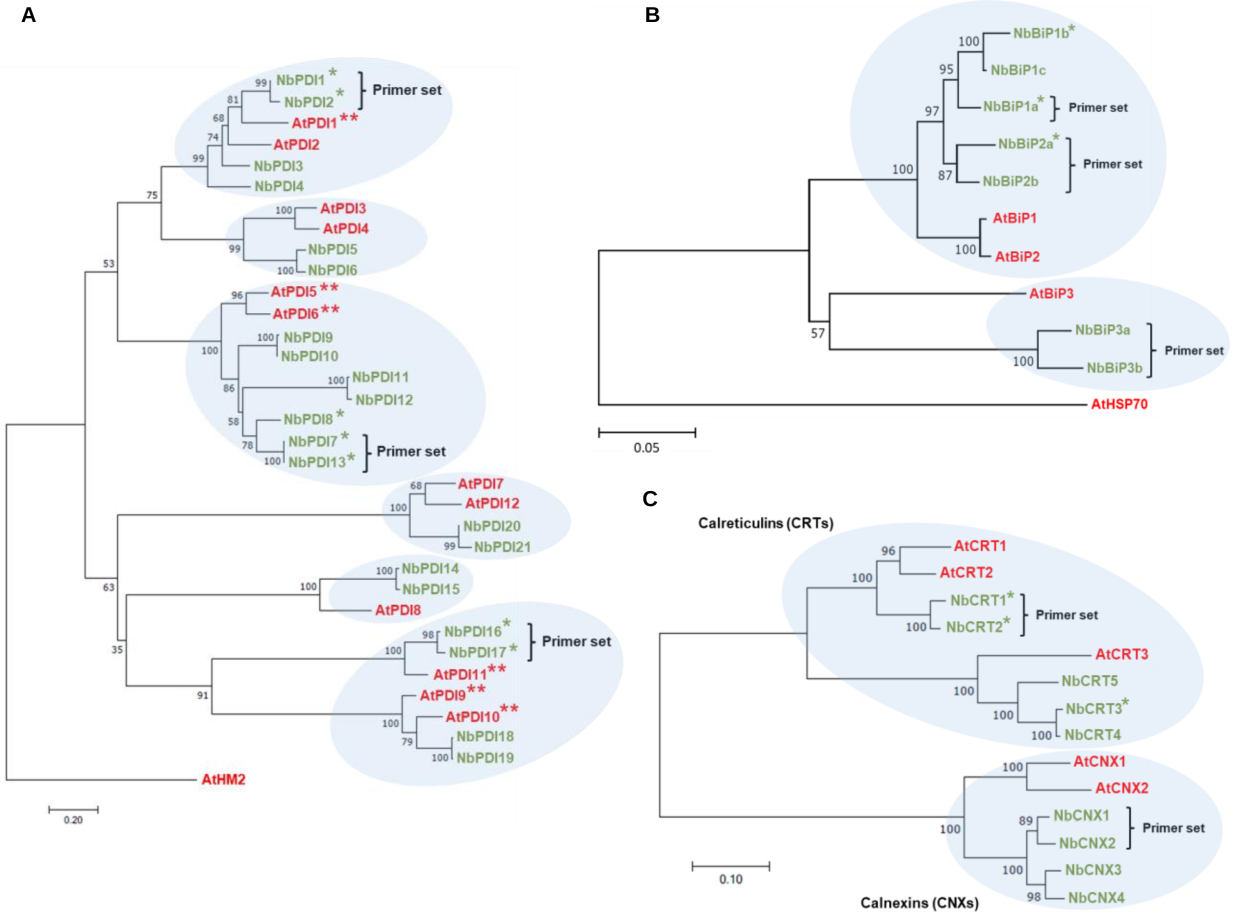

Figure S1. Phylogeny of *N. benthamiana* PDIs and ER-resident chaperones. (A) The genome of *N. benthamiana* was searched using full-length amino acid sequences of AtPDI1 (At3g54960) as a query. Full-length protein sequences that were retrieved were then aligned with ClustalW, using thioredoxin AtHM2 (At4g03520) as an outgroup. The resulting alignments were submitted to the MEGA5 software, and a neighbor-joining tree derived from 5,000 replicates was generated. Bootstrap values are indicated on the node of each branch. (B) For the phylogeny of BiPs, the same approach as above was employed, except that AtBiP1 (At5g28540) was used as a query to blast the genome and AtHSP70 (At3g12580) served as an outgroup. (C) For the phylogeny of CRT and CNX lectins, the same approach as above was employed, except that AtCRT1 (At1g56340) and AtCNX1 (At5g61790) were used as queries to blast the genome. The CNX cluster served as an outgroup for CRTs, and vice versa. *A. thaliana* (At) proteins are shown in red, while *N. benthamiana* (Nb) proteins are shown in green. Brackets highlight genes selected for RTqPCR. Single asterisks (*) highlight proteins identified by proteomics (Table 1; Hamel *et al.,* 2023). Double asterisks (**) denote AtPDIs involved in the UPR (Lu and Christopher, 2008). Gene model number corresponding to Arabidopsis and *N. benthamiana* proteins can be found in the Table S1.

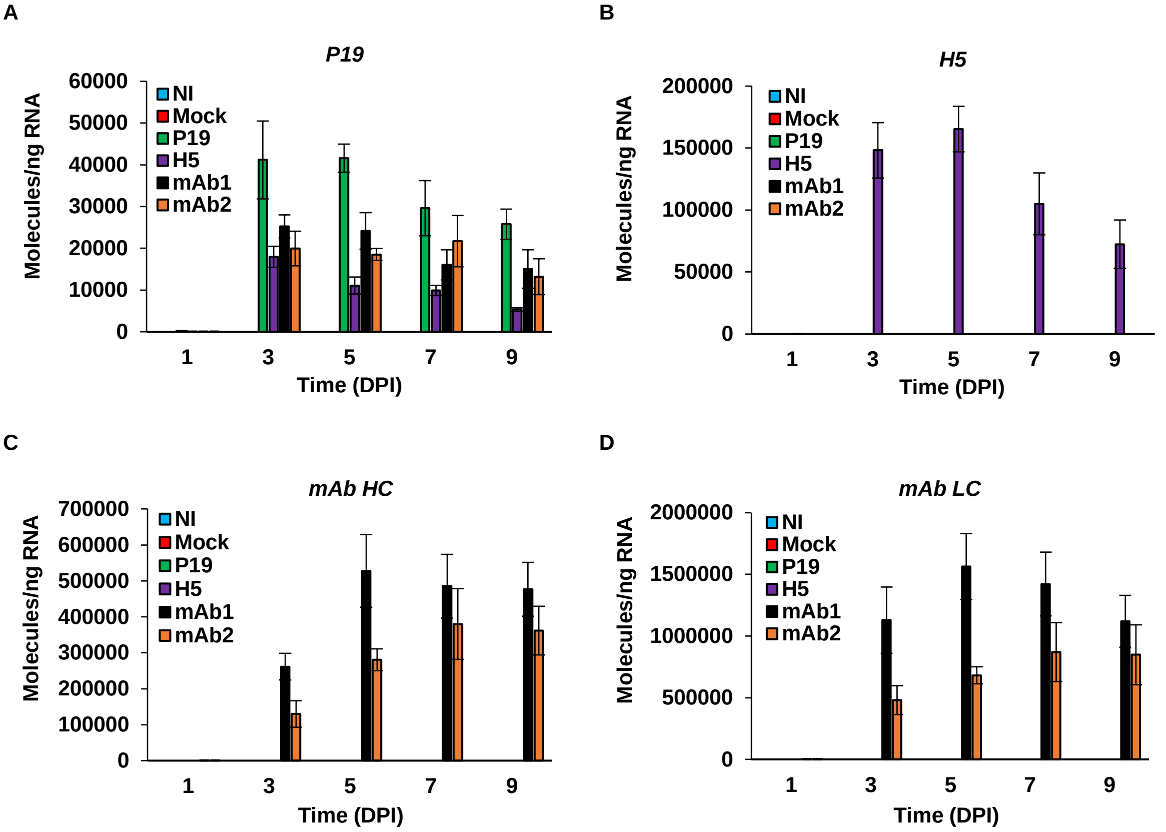

Figure S2. Expression of recombinant genes. Expression of recombinant genes *P19* (A), *H5* (B), *mAb HC* (C), and *mAb LC* (D), as measured by RTqPCR. For each time point in days post-infiltration (DPI), results are expressed in numbers of molecules per ng of RNA. Condition names are as follows: NI, non-infiltrated leaves; Mock, leaves infiltrated with buffer only; P19, agroinfiltrated leaves expressing P19 only; H5, agroinfiltrated leaves co-expressing P19 and H5; mAb1 (mAb2), agroinfiltrated leaves co-expressing P19 and monoclonal antibody 1 (monoclonal antibody 2).

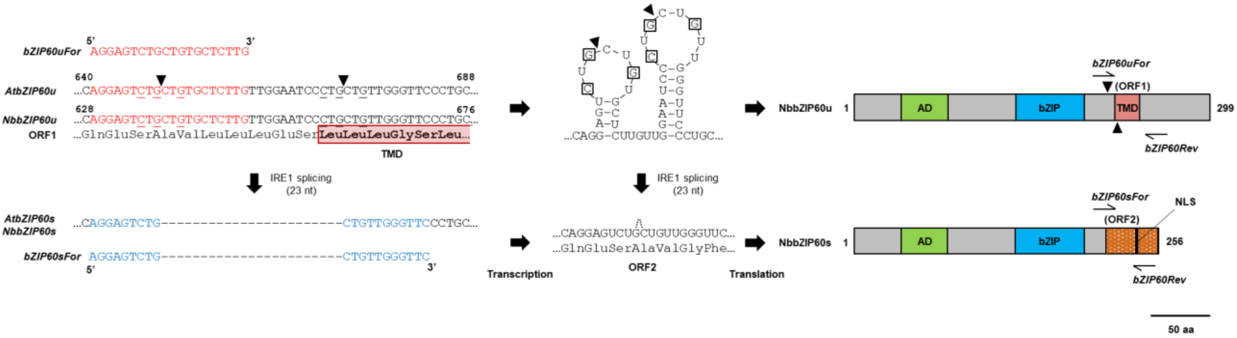

Figure S3. Primer design to assess unconventional splicing of *NbbZIP60*. Left section: nucleotide (nt) sequence alignment confirming conservation of unconventional splicing regions within *bZIP60* transcripts from *A. thaliana* (*AtbZIP60u*) and *N. benthamiana* (*NbbZIP60u*). Three-letter amino acid (aa) code of the corresponding open reading frame (ORF1) is shown and start of the transmembrane domain (TMD) is boxed in red. Conserved nt from the repeated CXGXXG motifs are underlined. As part of the stem-loop structures recognized by IRE1, these nt are essential for splicing of the mRNA. Black triangles indicate predicted mRNA splicing sites. After removal of a 23 nt intron, sequence of the spliced *bZIP60* (*bZIP60s*) is shown. Sequences from forward (For) primers used to discriminate spliced and unspliced versions of the transcripts are shown in blue (*bZIP60sFor*) and red (*bZIP60uFor*), respectively. Middle section: predicted stem-loops from the *bZIP60u* mRNA. Conserved nt from the repeated CXGXXG motifs are boxed and black triangles highlight predicted mRNA splicing sites. After removal of the 23 nt intron, nt and deduced aa sequences from the new open reading frame (ORF2) are shown. Right section: at-scale topology from predicted NbbZIP60u and NbbZIP60s proteins. The transcriptional activation domain (AD) is shown in green. The basic leucine zipper (bZIP) domain, which is required for protein dimerization and DNA-binding, is shown in blue. The TMD of NbbZIP60u is shown in red and the alternative C-terminus of NbbZIP60s in textured orange. The TMD is no longer translated, and this region now includes a nuclear localization signal (NLS) depicted in black. Based on corresponding transcripts sequences, relative position of the For and reverse (Rev) primers used for RTqPCR are shown. Rev primer was the same for both transcripts (*bZIP60Rev*). Adapted from Nagashima *et al.,* 2011.

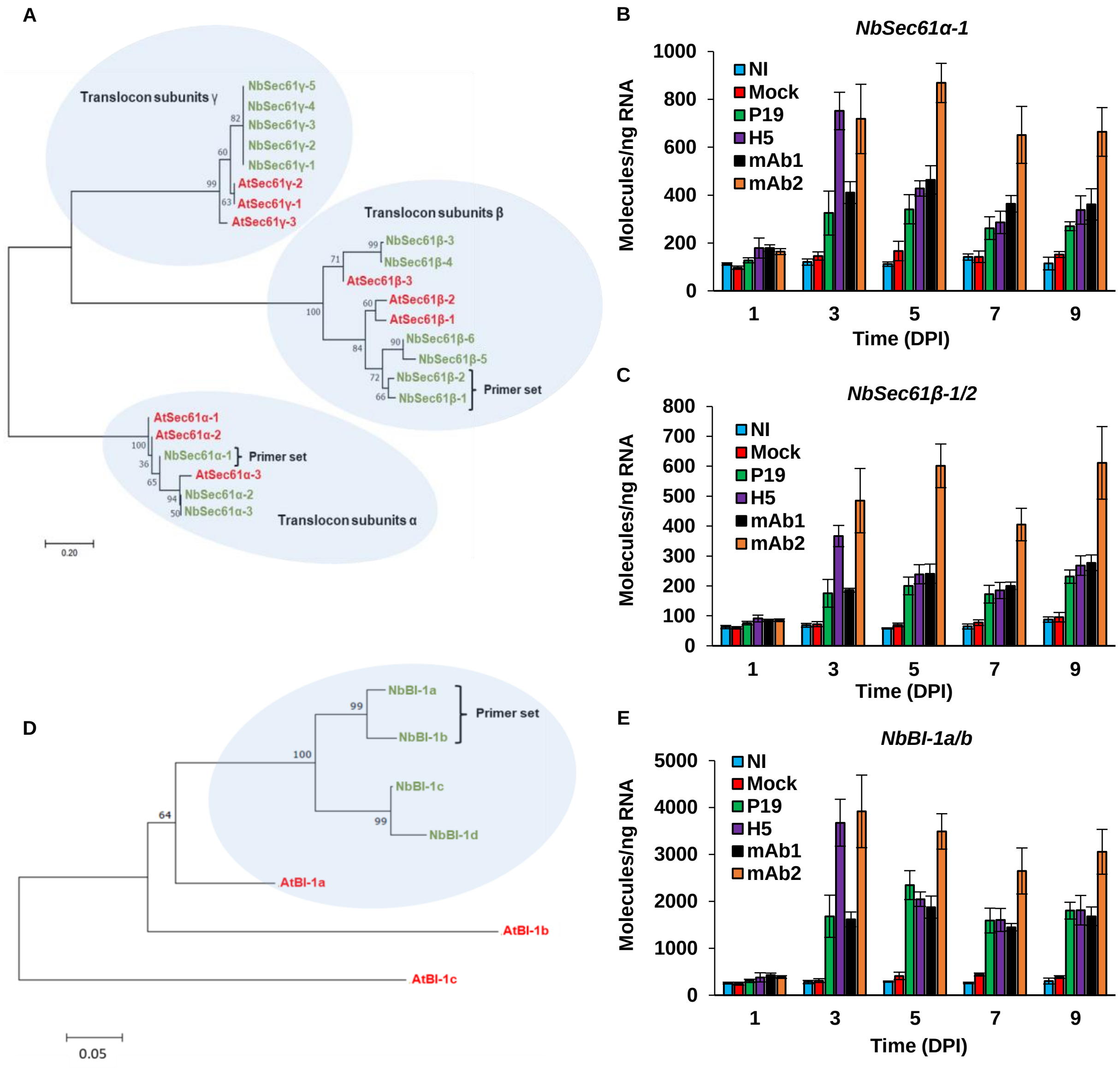

Figure S4. Phylogeny of other UPR proteins and expression of some of their corresponding genes. (A) For Sec61 proteins, the genome of *N. benthamiana* was searched using full-length amino acid sequences of AtSec61α-1 (At1g29310), AtSec61β-1 (At2g45070), and AtSec61γ-1 (Atg424920) as queries. Full-length protein sequences that were retrieved were next aligned with ClustalW. The different protein clusters served as outgroups for each other. The resulting alignments were submitted to the MEGA5 software, and a neighbor-joining tree derived from 5,000 replicates was generated. Bootstrap values are indicated on the node of each branch. *A. thaliana* (At) proteins are shown in red, while *N. benthamiana* (Nb) proteins are shown in green. Brackets highlight genes selected for RTqPCR. Gene model number corresponding to Arabidopsis and *N. benthamiana* proteins can be found in the Table S1. Expression of genes encoding Sec61 proteins NbSec61α-1 (B) as well as NbSec61β-1 and NbSec61β-2 (C), as measured by RTqPCR. For each time point in days post-infiltration (DPI), results are expressed in numbers of molecules per ng of RNA. (D) For the phylogeny of BI-1 proteins, the same approach as above was employed except that AtBI-1a (AT5G47120) was used as a query to blast the genome. Distant homolog AtBI-1c (At5g47130) served as an outgroup. Expression of *BI-1* genes *NbBI-1a* and *NbBI-1b* (E), as measured by RTqPCR. Condition names are as follows: NI, non-infiltrated leaves; Mock, leaves infiltrated with buffer only; P19, agroinfiltrated leaves expressing P19 only; H5, agroinfiltrated leaves co-expressing P19 and H5; mAb1 (mAb2), agroinfiltrated leaves co-expressing P19 and monoclonal antibody 1 (monoclonal antibody 2).

A

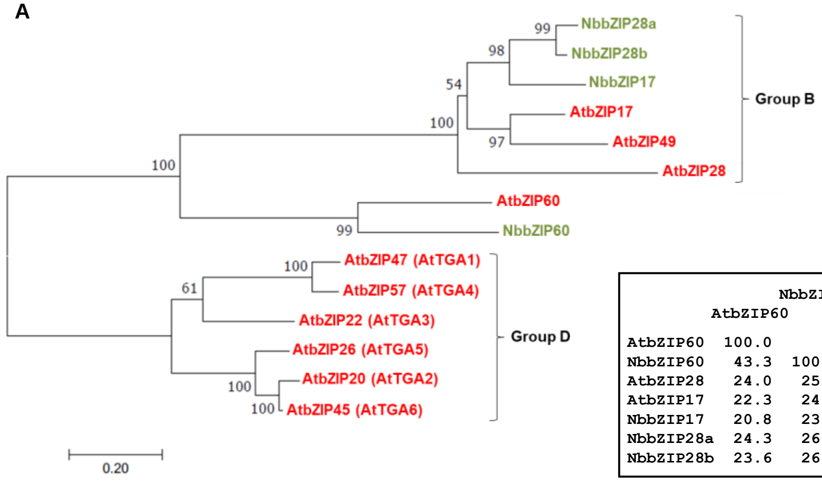

	AtbZIP60	NbbZIP60	AtbZIP28	AtbZIP17	NbbZIP17	NbbZIP28a	NbbZIP28b
AtbZIP60	100.0						
NbbZIP60	43.3	100.0					
AtbZIP28	24.0	25.1	100.0				
AtbZIP17	22.3	24.8	43.6	100.0			
NbbZIP17	20.8	23.2	40.0	50.7	100.0		
NbbZIP28a	24.3	26.7	46.2	48.9	65.2	100.0	
NbbZIP28b	23.6	26.3	47.1	49.9	65.2	91.7	100.0

B

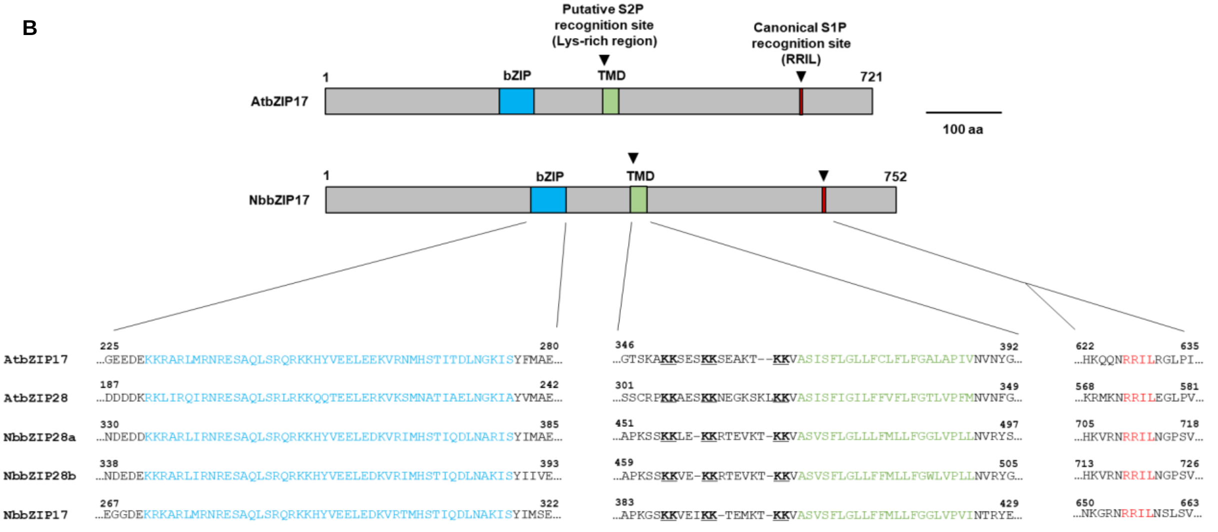

Figure S5. Phylogeny of UPR activating bZIPs and conservation of bZIP17 and bZIP28 in *N. benthamiana*. (A) The genome of *N. benthamiana* was searched using full-length amino acid sequences of AtbZIP17 (At2g40950), AtbZIP28 (At3g10800), and AtbZIP60 (At1g42990) as queries. Full-length protein sequences that were retrieved were then aligned with ClustalW, using a set of defense-related bZIPs from Arabidopsis as an outgroup (Group D; Stotz *et al.,* 2013). Resulting alignments were submitted to the MEGA5 software and a neighbor-joining tree derived from 5,000 replicates was generated. Bootstrap values are indicated on the node of each branch. *A. thaliana* (At) proteins are shown in red, while *N. benthamiana* (Nb) proteins are shown in green. Brackets highlight bZIP groups, as previously defined in Arabidopsis (Jakoby *et al.,* 2002). In this classification, bZIP60s remain unclassified. For UPR activating bZIPs, a percent identity matrix is shown on the right. Gene model number corresponding to bZIP17, bZIP28, and bZIP60 homologs from Arabidopsis and *N. benthamiana* can be found in the Table S1. (B) At-scale topology of AtbZIP17 and NbbZIP17 proteins. The basic leucine zipper (bZIP) domain, which is required for protein dimerization and DNA-binding, is shown in blue. The transmembrane domain (TMD) is shown in green. Canonical recognition site of Golgi-associated protease S1P is shown in red (RXXL motif). Clipping site of S1P and predicted clipping site of Golgi-associated protease S2P are depicted by black triangles. Conservation of functional regions within bZIP17 and bZIP28 homologs from Arabidopsis and *N. benthamiana* is depicted by a protein sequence alignment, with amino acids displayed using their one-letter code. S2P-mediated clipping is thought to occur within a lysine (Lys)-rich region located before the TMD and conserved Lys residues are bolded and underlined.

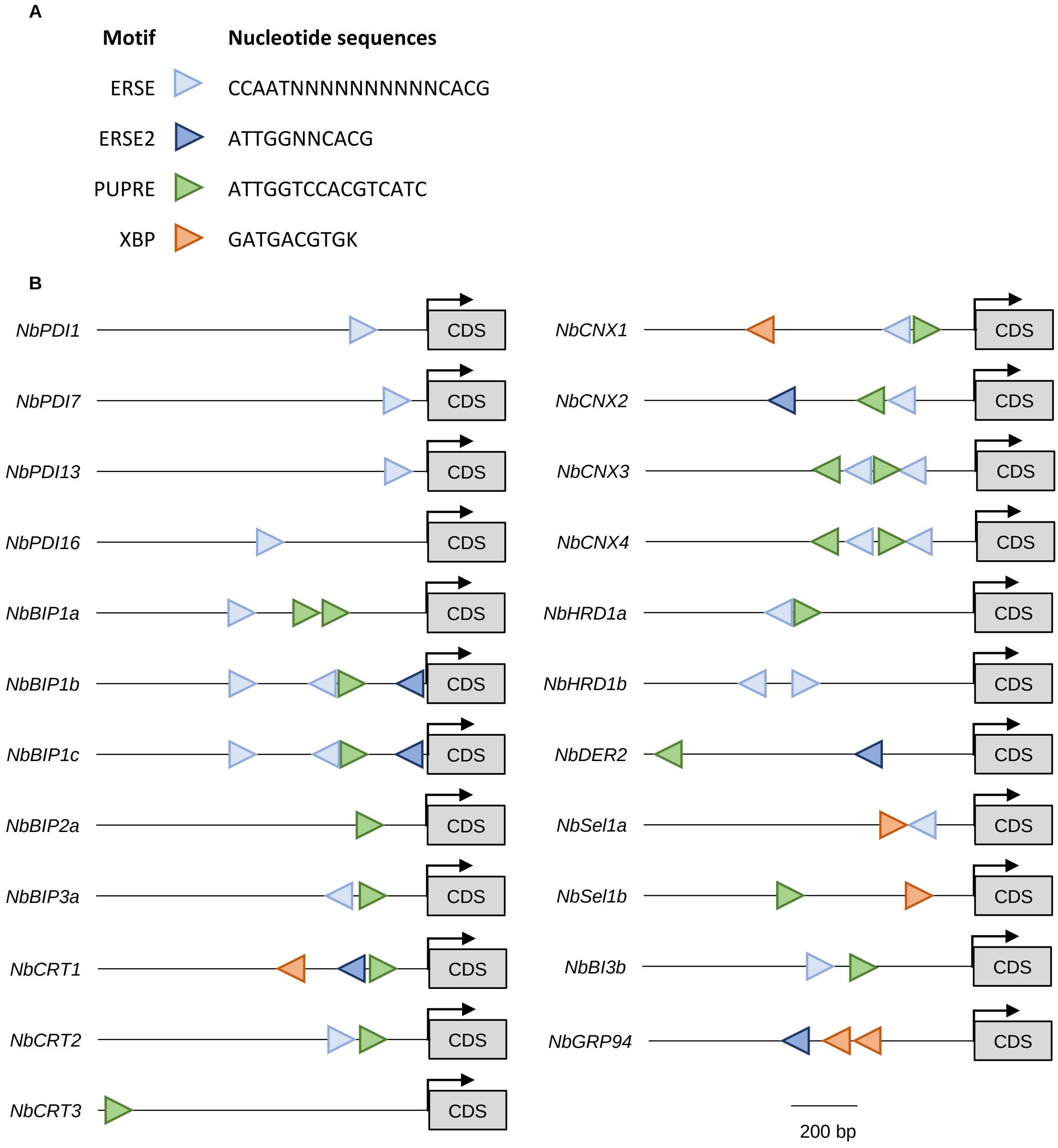

Figure S6. *Cis* regulatory elements in the promoter of UPR genes. (A) Consensus nucleotide sequences of *cis* regulatory elements that were examined. These motifs were previously linked to the UPR in plants (Iwata and Koizumi, 2005; Iwata *et al.*, 2008; Liu and Howell, 2016). Each motif is depicted by a color-coded triangle. "N" letters represent any nucleotide, while the "K" letter represents a guanine (G) or a thymine (T). (B) Using genome sequences from *N. benthamiana*, putative promoter regions from selected UPR genes were retrieved. For each gene, a 1000 base pairs (bp) region located upstream of the annotated start codon was employed and motif scanning was performed in both sequence orientations using the FIMO software (Grant *et al.*, 2011). Approximate location and orientation of each motif are indicated by color-coded triangles. Predicted start codon of each coding sequence (CDS) is highlighted using a black arrow.
